# Supplementary material for: Associations of remote mental healthcare with clinical outcomes: a natural language processing enriched electronic health record data study protocol
Source: BMJ Open. 2023 Feb 10;13(2):e067254. doi: 10.1136/bmjopen-2022-067254 (PMC9923317; doi:10.1136/bmjopen-2022-067254)
Supplement: Supplementary data [file bmjopen-2022-067254supp001.pdf]

Associations of remote mental healthcare with clinical outcomes: a natural language processing enriched electronic health record data study protocol.

Muhammad Shamim Ahmed[1], Daisy Kornblum[2], Dominic Oliver[1,3,4], Paolo Fusar-Poli[1,5], Rashmi Patel[2,6]

[1]Department of Psychosis Studies, King's College London, Institute of Psychiatry Psychology and Neuroscience, London, UK

[2] NIHR Maudsley Biomedical Research Centre, South London and Maudsley NHS Foundation Trust, London, UK

[3]Department of Psychiatry, University of Oxford, Oxford, UK

[4]NIHR Oxford Health Biomedical Research Centre, Oxford, UK

[5]Department of Brain and Behavioural Sciences, University of Pavia, Pavia, Italy

[6]Department of Psychological Medicine, King's College London, Institute of Psychiatry Psychology and Neuroscience, London, UK

Online Supplementary Material

Supplementary Table 1 Natural language processing applications

Application	Description	Example	Precision and recall
Symptoms and signs			
Aggression	Application to identify instances of aggressive behaviour in patients, including verbal, physical and sexual aggression.	‘Reported to be quite aggressive’	P=91% R=75%
Agitation	Application to identify instances of agitation.	‘Very agitated at present’	P=85% R=79%
Anergia	Application to identify instances of anergia.	‘Feelings of anergia’	P=95% R=89%
Anhedonia	Application to identify instances of anhedonia (inability to experience pleasure from activities usually found enjoyable).	‘No evidence of anhedonia’	P=93% R=86%
Apathy	Application to extract the presence of apathy.	‘Continues to demonstrate apathy’	P=93% R=86%
Arousal	Application to identify instances of arousal excluding sexual arousal.	‘Hyperaroused state”	P=89% R=91%
Bad Dreams	Application to identify instances of experiencing a bad dream.	‘Frequently has bad dreams	P=89% R=100%
Blunted Affect	Application to identify instances of blunted affect.	‘Affect remains very blunted’	P=100% R=80%
Circumstantiality	Application to identify instances of circumstantiality.	‘Loose associations and circumstantiality’	P=94% R=92%

Ahmed MS, *et al. BMJ Open* 2023; 13:e067254. doi: 10.1136/bmjopen-2022-067254

Concrete Thinking	Application to identify instances of concrete thinking.	'No evidence of concrete thinking'	P=84% R=41%
Delusions	Application to identify instances of delusions.	'Paranoid delusions'	P=93% R=85%
Derailment	Application to identify instances of derailment.	'He derailed frequently'	P=84% R=99%
Disturbed Sleep	Application to identify instances of disturbed sleep.	'Complains of poor sleep'	P=88% R=68%
Drowsiness	Application to identify instances of drowsiness.	'She has complained of feeling drowsy'	P= 80% R=100%
Echolalia	Application to extract occurrences where echolalia is present.	'Intermittent echolalia'	P=89% R=86%
Elation	Application to identify instances of elation.	'Mildly elated in mood'	P=94% R=97%
Emotional Withdrawal	Application to identify instances of emotional withdrawal.	'Patient withdrawn'	P=85% R=96%
Flight of Ideas	Application to extract instances of flight of ideas.	'Some flight of ideas.	P=91% R=94%
Formal Thought Disorder	Application to extract occurrences where formal thought disorder is present.	'Deteriorating into a more thought disordered state'	P=83% R=83%
Grandiosity	Application to extract occurrences where grandiosity is apparent.	'Reduction in grandiosity'	P=95% R=91
Guilt	Application to identify instances of guilt.	'No longer feels guilty'	P=83% R=83%
Hallucinations (All**)**	Application to identify instances of hallucinations.	'Doesn't appear distressed by his hallucinations'	P=84% R=98%
Hallucinations (Auditory)	Application to identify instances of auditory hallucinations non-specific to diagnosis.	'Experiencing auditory hallucinations'	P= 80%, R=84%
Hallucinations (Olfactory/Tactile/Gustatory)	Application to extract occurrences where hallucination ((Olfactory/Tactile/Gustatory) is present.	'Seems to be having olfactory hallucinations, in relation to her tactile hallucinations'	P=78%, R=68%
Hallucinations (Visual)	Application to extract occurrences where visual hallucination is present. Visual hallucinations may be due to a diagnosis of psychosis/schizophrenia or may be due to other causes, e.g., due to substance abuse.	'Experiencing visual hallucination'	P=91%, R=96%
Helplessness	Application to identify instances of helplessness.	'Ideas of helplessness'	P=93% R=86%
Hopelessness	Application to identify instances of hopelessness.	'Says feels hopeless'	P=90% R=95%
Hostility	Application to identify instances of hostility.	'Increased hostility and paranoia'	P=89%, R=94%

Ahmed MS, *et al. BMJ Open* 2023; 13:e067254. doi: 10.1136/bmjopen-2022-067254

Insomnia	Application to identify instances of insomnia.	'Complaining of insomnia'	P=89%, R=94%
Irritability	Application to identify instances of irritability.	'Became irritable'	P=100% R=83%
Loss of Coherence	Application to identify instances of incoherence or loss of coherence in speech or thinking.	'Patient was incoherent'	P=98% R=95%
Low Energy	Application to identify instances of low energy.	'Decreased energy'	P=72% R=67%
Mood Instability	This application identifies instances of mood instability.	'Expressed fluctuating mood'	P=100% R=70%
Mutism	Application to identify instances of mutism.	'Did not respond any further and remained mute'	P=91% R=75%
Negative Symptoms	Application to identify instances of negative symptoms.	'Diagnosis of schizophrenia with prominent negative symptoms'	P=86% P=95%
Nightmares	Application to identify instances of nightmares.	'Unsettled sleep with vivid nightmares'	P=89% R=100%
Paranoia	Application to identify instances of paranoia. Paranoia may be due to a diagnosis of paranoid schizophrenia or may be due to other causes, e.g., substance abuse.	'Vague paranoid ideation'	P=86%, R=94%
Passivity	Application to identify instances of passivity.	'Patient describes experiencing passivity'	P=89% P=100%
Persecutory Ideation	Application to identify instances of ideas of persecution.	'Marked persecutory delusions'	P=80% R=96%
Poor Appetite	Application to identify instances of poor appetite (negative annotations).	'Normal appetite'	P=83% R=71%
Poor Concentration	Application to identify instances of poor concentration.	'Finds it hard to concentrate'	P=84% R=60%
Poor Insight	Applications to identify instances of poor insight.	'Limited insight'	P=87% R=70%
Poor Motivation	This application aims to identify instances of poor motivation.	'Struggling with motivation'	P=95% R=38%
Poverty of Speech	Application to identify poverty of speech.	'Continues to display negative symptoms including poverty of speech'	P=87% R=85%
Poverty of Thought	Application to identify instances of poverty of thought.	'Evidence of poverty of thought'	P=95%, R=93%
Social Withdrawal	Application to identify instances of social withdrawal.	'Very isolated and socially withdrawn'	P=60% R=86%

Ahmed MS, *et al. BMJ Open* 2023; 13:e067254. doi: 10.1136/bmjopen-2022-067254

Stupor	Application to identify instances of stupor. This includes depressive stupor, psychotic stupor, catatonic stupor, dissociative stupor and manic stupor.	'Man with stuporous catatonia'	P=88% R=87%
Suicidal Ideation	Application to identify instances of suicidal ideation - thinking about, considering, or planning suicide.	'No suicidal ideation disclosed'	P=81% R=87%
Tangentiality	Application to identify instances of tangentiality.	'Evidence of tangential speech'	P=99% R=90%
Tearfulness	Application to identify instances of tearfulness.	'Appeared tearful'	P=100% R=94%
Thought Block	Application to identify instances of thought block.	'Showed some thought block'	P=91% R=75%
Thought Broadcast	Application to identify instances of thought broadcasting.	'Patient describes experiencing thought broadcasting'	P=86% R=92%
Thought Insertion	Application to identify instances of thought insertion.	'No thought insertion'	P=81% R=96%
Thought Withdrawal	Application to identify instances of thought withdrawal.	'Presence of thought withdrawal'	P=90% R=88%
Waxy Flexibility	Application to identify instances of waxy flexibility. Waxy flexibility is a psychomotor symptom of catatonia as associated with schizophrenia, bipolar disorder, or other mental disorders which leads to a decreased response to stimuli and a tendency to remain in an immobile posture.	'Presents as catatonic with waxy flexibility'	P=80% R=86%
Weight Loss	Application to identify instances of weight loss.	'Significant weight loss'	P=90% R=88%
Worthlessness	Application to identify instances of worthlessness.	'Feeling worthless'	P=88% P=86%
Contextual factors			
Asthma	Application to identify patients with diagnosis of asthma.	'Suffered from an asthma attack'	P=95% R=84%
Bronchitis	Application to identify patients with diagnosis of bronchitis.	'Past diagnosis: chronic obstructive airway disease'	P=85% R=48%
Cough	Application to identify instances of coughing.	'Denied any coughing or shortness of breath'	P=83% R=80%
Crohn's Disease	Application to identify patients with diagnosis of Crohn's disease.	'Has Crohn's disease'	P=94% R=78%
Hypertension	Application to identify patients with diagnosis of hypertension or high blood pressure.	'History of hypertension'	P=94% R=94%

Rheumatoid Arthritis	Application to identify patients with diagnoses of rheumatoid arthritis.	'Pain due to her rheumatoid arthritis'	R=91% R=86%
Amphetamine	To identify instances of amphetamine use.	'Has not used amphetamine for the last week'	P=80% R=84%
Cannabis	To identify instances of cannabis use.	'No cannabis use'	P=77% R=93%
Cocaine/Crack Cocaine	To identify instances of cocaine or crack cocaine use.	'He has stopped taking cocaine'	P=84% R=97%
MDMA	Application to identify instances of MDMA use.	'First took MDMA at the age of 15'	P=100% R=99%
Tobacco Smoking	This application distinguishes between people who are a) current smokers, b) current non-smokers (ever smoked) and c) non-smokers.	'Doesn't smoke'	P=81% R=74%
Lives Alone	Application to identify instances of living alone.	'Lives on her own'	P=77% R=83%
Domestic Violence;	Application to identify instances of domestic violence.	'Victim of DV'	P=86% R=93%
Loneliness	Application to identify instances of loneliness.	'Patient is lonely'	P= 87% R= 100%
Interventions			
Cognitive Behavioural Therapy (CBT)	An application to identify instances of delivered sessions of Cognitive Behavioural Therapy (CBT).	'Session of CBT'	P=99% R=82%
Family Intervention	The application identifies instances of family intervention delivery.	"Family therapy"	P=77% R=87%
Medication	The application identifies instances of medication prescription.	'Quetiapine'	Antipsychotics: P=97%-99%, R=39%
Outcomes and Clinical Status			
Blood Pressure (BP)	Application to identify instances of blood pressure scores in the format of overall score, systolic blood pressure score and diastolic blood pressure score.	'140/90mmHg'	P=98% R=96%
Body Mass Index (BMI)	Application to identify body mass index (BMI) scores.	'Body Mass Index is 22.9'	P=89% R=78%
HbA1c	Application to identify HbA1c results.	'HbA1c was 40mmol/mol'	P=89% R=93%
Mini-Mental State Examination (MMSE)	This app identifies MMSE scores.	'25/30'	P=93% R=94%
Diagnosis	Application to extract instances of diagnosis.	'Schizophrenia'	F20: P=100%, R=65%
Treatment-Resistant Depression	Application to identify instances of treatment-resistant depression.	'History of treatment resistant depression'	P=77% R=95%
Bradykinesia (Dementia)	To identify instances of bradykinesia in the context of dementia.	'Moderate bradykinesia'	P=91% R=84%

Tremor (Dementia)	Application to identify instances of tremor in patients with dementia.	‘With a degree of resting tremor’	P=83%, R=92%
