# Supplementary material for: Associations of remote mental healthcare with clinical outcomes: a natural language processing enriched electronic health record data study protocol
Source: BMJ Open. 2023 Feb 10;13(2):e067254. doi: 10.1136/bmjopen-2022-067254 (PMC9923317; doi:10.1136/bmjopen-2022-067254)
Supplement: Author's manuscript [file bmjopen-2022-067254.draft_revisions.pdf]

BMJ Open is committed to open peer review. As part of this commitment we make the peer review history of every article we publish publicly available.

When an article is published we post the peer reviewers' comments and the authors' responses online. We also post the versions of the paper that were used during peer review. These are the versions that the peer review comments apply to.

The versions of the paper that follow are the versions that were submitted during the peer review process. They are not the versions of record or the final published versions. They should not be cited or distributed as the published version of this manuscript.

BMJ Open is an open access journal and the full, final, typeset and author-corrected version of record of the manuscript is available on our site with no access controls, subscription charges or pay-per-view fees (http://bmjopen.bmj.com).

If you have any questions on BMJ Open's open peer review process please email info.bmjopen@bmj.com

BMJ Open

Natural Language Processing enriched electronic health record data studies to investigate the associations of remote mental health care with clinical outcomes in psychiatry.

Journal:	*BMJ Open*
Manuscript ID	bmjopen-2022-067254
Article Type:	Protocol
Date Submitted by the Author:	08-Aug-2022
Complete List of Authors:	Ahmed, Muhammad; Institute of Psychiatry Psychology and Neuroscience Division of Academic Psychiatry, Department of Psychosis Studies<br>Kornblum, Daisy; South London and Maudsley Mental Health NHS Trust, NIHR Maudsley Biomedical Research Centre<br>Oliver, Dominic; Institute of Psychiatry Psychology and Neuroscience Division of Academic Psychiatry, Department of Psychosis Studies<br>Fusar-Poli, Paolo; Institute of Psychiatry Psychology and Neuroscience Division of Academic Psychiatry, Department of Psychosis Studies<br>Patel, Rashmi; Institute of Psychiatry Psychology and Neuroscience Division of Academic Psychiatry, Department of Psychological Medicine; South London and Maudsley Mental Health NHS Trust, NIHR Maudsley Biomedical Research Centre
Keywords:	EPIDEMIOLOGY, Telemedicine < BIOTECHNOLOGY & BIOINFORMATICS, Health informatics < BIOTECHNOLOGY & BIOINFORMATICS, PSYCHIATRY

SCHOLARONE™
Manuscripts

Natural Language Processing enriched electronic health record data studies to investigate the associations of remote mental health care with clinical outcomes in psychiatry.

Muhammad Ahmed[1], Daisy Kornblum[2], Dominic Oliver[1], Paolo Fusar-Poli[1], Rashmi Patel[2,3]

[1]Department of Psychosis Studies, King's College London, Institute of Psychiatry Psychology and Neuroscience, London, UK

[2] NIHR Maudsley Biomedical Research Centre, South London and Maudsley NHS Foundation Trust, London, UK

[3]Department of Psychological Medicine, King's College London, Institute of Psychiatry Psychology and Neuroscience, London, UK

Correspondence to:

Rashmi Patel

bmj@rpatel.co.uk

Word count: 3,545

Keywords: natural language processing, electronic health records, remote mental healthcare

For peer review only

For peer review only - http://bmjopen.bmj.com/site/about/guidelines.xhtml

Natural Language Processing enriched electronic health record data studies to investigate the associations of remote mental health care with clinical outcomes in psychiatry.

ABSTRACT

Introduction People often experience significant difficulties in receiving mental healthcare due to insufficient resources, stigma and lack of access to community mental health services. Remote care technology has the potential to overcome these barriers and augment in-person care by reducing time spent travelling to see patients and increasing frequency of contact with patients who require intensive support. However, the safe delivery of remote mental healthcare requires evidence on which aspects of care are suitable for remote delivery and which are better served by in-person contacts. We aim to generate evidence on the associations of remote mental healthcare with clinical outcomes using electronic health record (EHR) data enriched with natural language processing (NLP).

Methods and analysis De-identified EHR data, derived from the South London and Maudsley (SLaM) NHS Foundation Trust Biomedical Research Centre (BRC) Case Register, will be extracted using the Clinical Record Interactive Search tool for all patients receiving mental healthcare between 1st January 2019 and 31st March 2022. First, data on a retrospective, longitudinal cohort of around 80,000 patients will be analysed using multivariable Cox regression to compare outcomes of remote vs in-person assessments. Second, NLP techniques will be applied to around 5 million documents to analyse the content of remote vs in-person assessments.

Ethics and dissemination The SLaM BRC Case Register and CRIS tool have received ethical approval as a de-identified dataset for secondary mental health research analyses from Oxfordshire REC C (Ref: 18/SC/0372). The study has received approval from the SLaM CRIS Oversight Committee (chaired by a patient representative) which reviews and approves applications to extract and analyse the data. Study findings will be disseminated through peer-reviewed, open access journal articles and service user and carer advisory groups.

For peer review only

For peer review only - http://bmjopen.bmj.com/site/about/guidelines.xhtml

Natural Language Processing enriched electronic health record data studies to investigate the associations of remote mental health care with clinical outcomes in psychiatry.

Article summary

Strengths and limitations of this study

- The use of electronic health record data will enable comparisons between remote vs in-person assessments in a large sample of NHS patients with mental health disorders.
- Natural language processing models will enable analysis of free text data to ascertain differences in clinical presentation, assessment and outcomes between patients receiving remote and in-person assessments in electronic health records.
- The large sample size is representative of a real-world clinical practice.
- Routinely recorded data will be investigated for secondary research purposes. However, data on clinical outcomes may be missing or inaccurate. Data availability relies on the recording of accurate data at the point of clinical contact.
- The study will analyse secondary mental health data which does not include data from primary care.

For peer review only

For peer review only - http://bmjopen.bmj.com/site/about/guidelines.xhtml

INTRODUCTION

Remote technologies such as phone, video calls, text messages and e-mail allow patients and clinicians to communicate with each other when it is not possible to meet in-person. The COVID-19 pandemic accelerated the implementation of remote care technology in mental health with services often using remote technology to maintain clinical contact and deliver care[1,2]. Although restrictions resulting from the pandemic have eased, remote technology is likely to continue to be used and remain a useful adjunct to conventional in-person consultation[3].

Access to conventional mental healthcare may be hindered due to lack of resources, stigma, and lack of access to community mental health services[1]. Remote care technology has the potential to overcome these barriers and augment in-person care[4]. Additionally, remote technology has proved surprisingly popular with both clinicians and patients[3]. However, some patients are likely to find remote technology less helpful than others. They may lack necessary equipment and reliable internet connectivity, be unfamiliar and uncomfortable with the technology, or have mental disorders that affect their motivation and cognitive functioning making it difficult to use. Patients, carers and clinicians may also have concerns about privacy and security[5]. The potential value of this approach in mental health care has yet to be systematically evaluated and the evidence base to support best practice is sparse. The potential benefits of remote care must be balanced against the risk that its use might, in some situations, have adverse effects on quality of care, patient satisfaction, and clinical outcomes. There is a pressing need to formally evaluate the impact of remote care technology on mental healthcare so that potential barriers to accessing remote care can be identified, its benefits maximised, and potential risks minimised.

Electronic health record (EHR) data could be used to address this gap in understanding. EHRs have been widely adopted in NHS healthcare services[6]. Information is stored as both structured data, which includes categorical data such as demographics, prescribed medications, and diagnoses, and, to a greater extent, unstructured data such as free text notes which tend to be written in narrative format by clinical or administrative staff. Unstructured data may contain details on symptoms, diagnoses, and treatment plans that are otherwise not available in structured data fields. EHRs not only support individual patient care but also have the potential to support largescale research through the analysis of de-identified clinical data[7]. Natural language processing (NLP) techniques can be used to automatically and rapidly extract clinical information from unstructured free text clinical notes and correspondence to assess the associations of clinical factors,[7,8] environmental factors[9] and treatments[10,11] with clinical outcomes.

We aim to investigate differences in clinical outcomes associated with remote vs in-person assessments from a large sample of patients receiving care for mental disorders by applying NLP to extract data from de-identified EHR records and comparing clinical outcomes between in-person and remote treatment pathways.

METHODS AND ANALYSIS

Study aims and design

We will conduct two studies. First, we aim to conduct a retrospective, longitudinal cohort study to examine the potential associations between remote care and clinical outcomes and to test the hypothesis that (a) use of remote care will be lower in patients with poor motivation, and (b) remote care use will be more frequent in the treatment of less severe disorders, such as mild depression and anxiety, whereas in-person care will be more frequent in the treatment of more severe disorders, such as psychotic and bipolar mood disorders.

Second, an NLP study which will compare clinical documentation between in-person appointments and remote care appointments. We hypothesise that (a) the documentation of diagnosis, symptoms and treatments will be similar for remote and in-person care, (b) documentation of clinical signs (as

For peer review only - http://bmjopen.bmj.com/site/about/guidelines.xhtml

part of mental state examination) will be less frequent with remote than with in-person care, and (c) use of remote care will be associated with fewer documented episodes of suicidal thoughts. We hypothesise that differences between remote and in-person care will be less evident when remote assessment involves video, as opposed to audio or text.

Study setting

The data used in this study will be obtained from the South London and Maudsley (SLaM) National Health Service Foundation Trust. SLaM is one of the largest providers of specialist mental healthcare in Europe and provides more than 230 services, including specialist psychosis services, inpatient wards, community and outpatient services, within its catchment serving approximately 1.3 million residents in the London boroughs of Lambeth, Southwark, Lewisham and Croydon. SLaM treats over 5,000 inpatients and 40,000 outpatients per year. The Trust has implemented a fully electronic health record system since 1 April 2006 and the SLaM NIHR Biomedical Research Centre (BRC) Case Register contains the clinical records of 417,482 patients (accurate on 08/02/2022)[12].

Data source

Since April 2006, SLaM has utilised an electronic psychiatric clinical records system, the electronic Patient Journey System (ePJS), to record instances of patients accessing or interacting with health services. The SLaM BRC Case Register extracts anonymised clinical data from ePJS including structured fields (for demographic, diagnostic and medication data) and de-identified unstructured free text fields from case notes and correspondence. Clinical information is documented by healthcare professionals during the provision of mental healthcare to patients and includes history, mental state examination, diagnostic formulation and care plans. Healthcare professionals who document clinical data include psychiatrists, psychologists, nursing staff, care coordinators and allied healthcare professionals. A patient-led oversight committee considers all proposed research before access to the anonymised data is permitted and access to data is restricted to honorary or substantive employees of SLaM[13].

Data for this study will be obtained from the SLaM BRC Case Register using the Clinical Record Interactive Search tool (CRIS), a bespoke Microsoft Structured Query Language (SQL) database query and assembly tool that allows authorised researchers to extract and recode data from the SLaM BRC Case Register. Data are obtained from structured electronic health record fields and from unstructured free-text documentation using natural language processing (NLP)[6]. Data analysis will commence on 1st October 2022.

Natural language processing

To maximise ascertainment of clinical data in this study, data will be obtained from both structured fields and unstructured free-text clinical entries (using NLP). As EHRs provide limited structured information, a suite of NLP models has been developed to enrich the SLaM BRC Case Register by allowing clinical information to be extracted from unstructured free text in the BRC Case Register, including assessments, reviews and correspondence documented as unstructured free text. These yield complementary information on diagnoses, symptomatology and other patient characteristics[14].

Their performance of an NLP model is measured through precision and recall. Precision refers to the number of true positive instances that the NLP model has identified, divided by the total number of instances retrieved by the NLP model (both true positive and false positive instances). Recall refers to the number of true positive instances identified by the NLP model, divided by the total number of existing true positives in the entire sample. Precision and recall statistics for the NLP models employed in this project are supplied in supplementary table 1. The full library of NLP models available within CRIS and details of their development are provided on the CRIS website[15].

For peer review only - http://bmjopen.bmj.com/site/about/guidelines.xhtml

Study population

Data will be extracted for all patients of all ages who had an active episode of care from 1st January 2019 until 31st March 2022. An active episode of care is defined as at least one clinical contact with a healthcare professional with SLaM services during the study period.

Index dates

Data extracted for both studies will be from the first active episode of care after 1st January 2019 to 31st March 2022. This time-period was selected to provide data on clinical assessments prior to and during the COVID-19 pandemic (declared by the World Health Organisation in March 2020)[16], and on follow-up data relating to longitudinal clinical outcomes.

The index date for both studies will be the 1st of January 2019. For the longitudinal cohort study, all patients who have an active episode of care during 2019 and during 2020 will be considered. Follow-up data will be obtained from 1st January 2021 to 31st March 2022. For the NLP study, data on clinical assessments and clinical outcomes will be considered from 1st January 2019 to 31st March 2022.

Data extraction

Data derived from the SLaM BRC Case register will be interrogated using Structured Query Language (SQL), a standardised language for accessing and manipulating relational databases. SQL enables the application of queries to gather data from different parts of the SLaM BRC Case Register at the individual patient level. The use of SQL with electronic health record databases allows researchers to identify specific patient samples and extract relevant longitudinal clinical data. Demographic data on age, gender, ethnicity, marital status, occupation, housing status, multiple deprivation score and date of death, and clinical data on psychiatric disorder diagnosis and comorbid diagnoses will be extracted using SQL.

Data on type of clinical event attended (in-person vs remote including did not attend), type of remote event (video, audio or text), number of hospital admissions (sections and voluntary), date of hospital admission, date of discharge, number of A&E Liaison Psychiatry care episodes, number of Crisis Resolution Home Treatment Team (CRHTT) care episodes and whether psychotropic medications (antidepressants, antipsychotics and mood stabilisers) were prescribed will be extracted to support descriptive and inferential analyses.

Patient and public involvement

The study was developed in collaboration with the SLaM CRIS Service User and Carer Advisory Group (SUCAG), which includes patients and carers who have experience of SLaM mental healthcare and experience of supporting EHR data research studies. The study has received approval from the SLaM CRIS Oversight Committee (chaired by a patient representative) which reviews and approves applications to extract and analyse the data. The CRIS infrastructure was designed and is managed with ongoing patient and carer input to ensure all research projects comply with data governance, legal and ethical guidelines. Further details are available on the SLaM CRIS website[17].

Defined variables

The predictor variables and covariates for both the Cohort and NLP studies will be the same.

Predictors

The type of clinical event attended by a patient, categorised as in-person, remote (video call, phone, letter, instant or short message service (SMS) text message, e-mail), or did not attend (DNA). A

For peer review only - http://bmjopen.bmj.com/site/about/guidelines.xhtml

composite predictor variable will be generated as the proportion of all appointments (denominator) that are remote care appointments (numerator) between 1st January 2019 until 31st March 2022 as a measure of exposure to remote care. Also, a variable indicating the presence of documented poor motivation will be generated from unstructured text using NLP.

Covariates

Age, gender, ethnicity, occupation, marital status, psychiatric disorder ICD-10 diagnosis, housing status, and index of social deprivation will be obtained through a data linkage with the London Data Store[18] at Lower Super Output Area (LSOA) level.

Covariate categories will be coded according to NHS Data Dictionary definitions[19]. All predictor and covariate variables will be obtained from structured EHR data fields except for psychiatric disorder diagnosis, marital status and housing status which will be obtained from both structured and unstructured data using NLP using methods described subsequently.

Outcome measures

Cohort study

Primary outcome variable: psychiatric hospital admissions categorised as voluntary or involuntary (under a section of the UK Mental Health Act). Psychiatric hospital admission was chosen as the primary outcome variable to compare illness severity associated with the type of clinical event attended by a patient.

Secondary outcome variables: A&E Liaison Psychiatry care episodes, Crisis Resolution Home Treatment Team (CRHTT) care episodes, psychotropic medication prescribing (antidepressants, antipsychotics and mood stabilisers), ICD-10 psychiatric disorder diagnosis, attendance/non-attendance of in-person appointments, attendance/non-attendance of remote care appointments, and mortality as recorded in EHR data and through a pre-existing data linkage with the Office for National Statistics (ONS) to obtain data on cause of death including natural causes, suicide or open coroners' verdict, or other unnatural causes (unlawful killing, accident or misadventure).

All outcome variables will be obtained from structured EHR data fields.

NLP Study

Outcome variable: difference in frequency in documentation of diagnosis, symptoms, clinical signs (as part of mental state examination), treatments and episodes of suicidal thoughts between clinical documents related to in-person appointments compared to documents related to remote care appointments.

Statistical analysis

Data analysis will be performed using R statistical computing and graphics software.

Cohort study

1. Descriptive statistics

Descriptive statistics will be obtained for demographic variables, including age, gender, ethnicity, marital status, housing status, occupation and multiple deprivation score. Frequencies and percentages will be obtained for categorical variables and mean and standard deviations will be obtained for continuous variables.

2. Multivariable logistic regression analyses

For peer review only - http://bmjopen.bmj.com/site/about/guidelines.xhtml

Multivariable logistic regression will be used to analyse the potential association between type of We will generate an evidence base to support safe and effective remote mental healthcare that can positively impact clinical practice and service delivery. The ability to support greater access to mental healthcare through remote technology would reduce barriers and costs in providing care to people who would benefit from this approach while increasing capacity to provide in-person care to those who would prefer or cannot be safely treated remotely. This would enable more patients to receive care than is currently possible thereby improving clinical outcomes, quality of life and functioning for a greater number of people suffering with mental illness as well as reduced burden on carers and cost-effectiveness of mental healthcare provision.
clinical event attended and (i) psychiatric hospital admission; (ii) A&E Liaison Psychiatry care episodes; (iii) Crisis Resolution Home Treatment Team (CRHTT) care episodes; (iv) primary psychiatric diagnosis; (v) comorbid diagnosis; (vi) antidepressant, antipsychotic and mood stabiliser prescription and (vii) mortality.

Multivariable logistic regression will be used to analyse the potential association between (i) poor motivation and clinical event attendance; (ii) primary psychiatric disorder diagnosis and clinical event attendance; and (iii) comorbid psychiatric disorder diagnosis and clinical event attendance.

3. Multivariable cox regression analyses

Multivariable Cox regression will be used to analyse the potential association between type of clinical event attended and time to (i) voluntary psychiatric hospital admission; (ii) involuntary psychiatric hospital admission; (iii) A&E Liaison Psychiatry care episodes; (iv) Crisis Resolution Home Treatment Team (CRHTT) care episodes; (v) primary psychiatric disorder diagnosis; (vi) comorbid psychiatric disorder diagnosis; (vii) antidepressant, antipsychotic and mood stabiliser prescription and (viii) mortality.

NLP Study

NLP will be used to obtain data using applications previously developed by RP in collaboration with the NIHR Maudsley Biomedical Research Centre (BRC)[7,8,20,21]. Supplementary table 1 summarises the NLP applications and full details of the specification and precision statistics are available from the CRIS NLP Library[15].

NLP data will be obtained for each unstructured document representing an in-person or remote care event. The number of in-person vs remote care documents containing each NLP-derived construct will be compared using Chi-squared tests and logistic regression (predictor variable: type of clinical contact; outcome variable: presence of NLP-derived construct).

Sample size

As sample sizes of number of patients and number of documents in the SLaM BRC Case Register can be estimated from previous years' data, the pwr package (R v4.0.2) was used to estimate the minimal detectable effect size (MDE) for the cohort study and NLP study analyses using a significance level of 0.001 and power of 0.8 as follows:

Cohort study: MDE Cohen's w (sample size: 78,805 active patients in 2018 and 2019; df: 20): 0.022

NLP Study: MDE Cohen's w (sample size: 5,083,863 documents in 2018 and 2019; df: 100): 0.004

This indicates the data available will be well powered to detect small changes in effect size.

Missing data

For peer review only - http://bmjopen.bmj.com/site/about/guidelines.xhtml

Natural Language Processing enriched electronic health record data studies to investigate the associations of remote mental health care with clinical outcomes in psychiatry.

Based on previous studies, data on the predictor variable, age, gender and all outcome variables will be complete within the SLaM BRC Case Register. Where there are missing data for other covariates, two analyses will be conducted: a full sample analysis including missing data as a category in regression analyses and a complete case analysis dropping individuals who have one or more missing covariates.

Ethics and dissemination

The SLaM BRC Case Register and CRIS tool have received ethical approval as a de-identified dataset for secondary mental health research analyses from Oxfordshire REC C (Ref: 18/SC/0372). The study has received approval from the SLaM CRIS Oversight Committee (chaired by a patient representative) which reviews and approves applications to extract and analyse the data. The CRIS infrastructure was designed and is managed with ongoing patient and carer input to ensure all research projects comply with data governance, legal and ethical guidelines. Further details are available on the SLaM CRIS website[17]. Study findings will be disseminated through peer-reviewed journals and published open access to ensure there are no limitations to readership and access to their contents are as widely available as possible.

DISCUSSION

The COVID-19 pandemic has accelerated the implementation of remote care technology in mental health with a rapid shift towards the use of electronic written, audio and video communication[1,2]. However, this process was reactive, unplanned and, therefore, could not be informed by evidence on best practice. Nevertheless, remote technology has proved surprisingly popular with both clinicians and patients, and it is likely that it will continue to be used after the pandemic is over[4].

In a large NHS mental healthcare Trust, during the first wave of the COVID-19 pandemic there was a 23% fall in daily inpatient numbers between 1st January and 31st May 2020[22], a period associated with a substantial increase in remote care appointments[1]. Liaison psychiatry services experienced the largest reduction in caseloads across all of the Trust's mental healthcare teams[2]. However, these findings are confounded by the impact of the COVID-19 pandemic on changes in help-seeking behaviour due to travel restrictions and alterations in hospital bed configurations. Further research using multivariable regression techniques is necessary to adjust for potentially confounding factors.

People often experience significant difficulties in accessing conventional mental healthcare due to insufficient resources, stigma and lack of access to community mental health services[1]. Remote care technology has the potential to overcome these barriers and augment in-person care[4]. Remote video appointments are associated with higher attendance rates than in-person appointments, which may reflect a reduction in time and travel constraints[23]. The ability to observe family interactions in a home setting is a potential advantage for remote mental health consultations[24]. For older adults, the success of remote monitoring interventions to monitor and improve their health depends on the support and training they received in the use of the technology[25]. Concerns about the effectiveness of remote care technology have also been highlighted in services for people with intellectual disability and neurodevelopmental disorders[26]. Therefore, new research should include data from a wide range of patients who may use remote technology in different ways. We will address this need in this study by including data from one of the largest providers of mental healthcare in Europe that covers four socioeconomically diverse South London boroughs, alongside tertiary referrals from the rest of London and the United Kingdom. As such, the study population should encompass the requisite diversity in technological literacy and socioeconomics to provide results that are generalisable to other healthcare settings.

Remote care can also have benefits for clinical teams. As the demand for mental health services increases, clinicians are increasingly required to manage larger caseloads. In mental healthcare,

For peer review only - http://bmjopen.bmj.com/site/about/guidelines.xhtml

remote clinical assessment enables diagnostic accuracy comparable to in-person care[27]. There is no significant difference in accuracy between in-person and videoconferencing assessments when using standardised diagnostic instruments[28]. However, the routine use of standardised instruments is not reflective of real-world clinical practice. Additionally, compared to conventional care alone, remote care is associated with better treatment adherence[29], fewer hospital admissions and fewer days in hospital[23], and reduced severity of positive psychotic and manic symptoms[30]. However, negative psychotic symptoms are not improved by additional telephone support[30]. To ensure ecological validity, this study will focus on associations between remote assessments with real-world clinical outcomes, such as hospitalisation, instead of standardised diagnostic or psychometric instruments.

Safe delivery of remote mental healthcare requires evidence on which aspects of care are suitable for remote delivery and which are better served by in-person contact. Little is currently known about which patients are likely to benefit from remote care approaches and which patients will continue to need ongoing in-person care. Furthermore, certain patient groups who may benefit from remote care may be unable to access it. They may lack necessary equipment and reliable internet connectivity, be unfamiliar and uncomfortable with the technology, or have mental disorders that affect their motivation and cognitive functioning making it difficult to use. By investigating the impact of variables such demographic and clinical factors on non-attendance for remote assessments, we may be able to better understand the profiles of patients who may require additional support to benefit from remote healthcare. Our research will provide important evidence for mental health services on how best to implement remote care technology to ensure equity of access and maximise the number of patients who could benefit from this approach while ensuring that those who need in-person care are not disadvantaged.

Conclusion

There is a pressing need to formally evaluate the impact of remote care technology on mental healthcare so that potential barriers to accessing remote care can be identified, its benefits maximised, and potential risks minimised. Our research will provide evidence pertaining to identification of: i) patient groups that will most benefit from remote care, and those in whom in-person care remains a better approach; ii) types of clinical activity for which remote consultation is most suited; iii) risks associated with reducing in-person consultations; (iv) differences in clinical outcomes associated with remote vs in-person consultations.

For peer review only - http://bmjopen.bmj.com/site/about/guidelines.xhtml

Natural Language Processing enriched electronic health record data studies to investigate the associations of remote mental health care with clinical outcomes in psychiatry.

Data management and confidentiality

To comply with all necessary data protection regulations, EHR data will be stored and analysed using encrypted KCL/SLaM workstations and virtual machines hosted in a UK-based Microsoft Azure datacentre.

Twitter

Rashmi Patel @RPatelDr

Contributors The studies were conceived and designed by RP. Protocol preparation, literature review, statistical analysis plan and manuscript preparation was performed by MA, DK, DO, PFP and RP. All authors approved the final manuscript.

Funding RP has received funding from an NIHR Advanced Fellowship (NIHR301690), a Medical Research Council (MRC) Health Data Research UK Fellowship (MR/S003118/1) and an Academy of Medical Sciences Starter Grant for Clinical Lecturers (SGL015/1020) supported by the Wellcome Trust, MRC, British Heart Foundation, Arthritis Research UK, the Royal College of Physicians and Diabetes UK.

Disclaimer This project is funded by the National Institute for Health Research (Advanced Fellowship), grant number NIHR301690. The views expressed are those of the authors and not necessarily those of the NHS, the NIHR or the Department of Health. The funders had no role in the design and conduct of the study; collection, management, analysis and interpretation of the data; preparation, review or approval of the manuscript and decision to submit the manuscript for publication.

Competing interests All authors have completed the ICMJE uniform disclosure form at www.icmje.org/coi_disclosure.pdf and declare: RP has received funding from Janssen and Holmusk, outside the submitted work. The other authors declare no competing interests.

Patient and public involvement The study was developed in collaboration with the SLaM CRIS Service User and Carer Advisory Group (SUCAG), which includes patients and carers who have experience of SLaM mental healthcare and experience of supporting EHR data research studies. The study has received approval from the SLaM CRIS Oversight Committee (chaired by a patient representative) which reviews and approves applications to extract and analyse the data. The CRIS infrastructure was designed and is managed with ongoing patient and carer input to ensure all research projects comply with data governance, legal and ethical guidelines. Further details are available on the SLaM CRIS website[17].

Patient consent for publication Not applicable.

Ethics approval This study involves data from human participants and was approved by Oxfordshire REC C (Ref: 18/SC/0372). The CRIS data resource received ethical approval as a deidentified dataset for secondary mental health research analyses from Oxfordshire REC C (Ref: 18/SC/0372). The study was approved by the SLaM BRC CRIS oversight committee. Consent is not required to analyse the deidentified dataset for approved research studies. Patients may opt-out of inclusion in the deidentified dataset.

Provenance and peer review Not commissioned; externally peer reviewed.

Data availability statement No additional data are available.

Supplemental material This content has been supplied by the author(s). It has not been vetted by BMJ Publishing Group Limited (BMJ) and may not have been peer-reviewed. Any opinions or recommendations discussed are solely those of the author(s) and are not endorsed by BMJ. BMJ

For peer review only

For peer review only - http://bmjopen.bmj.com/site/about/guidelines.xhtml

disclaims all liability and responsibility arising from any reliance placed on the content. Where the content includes any translated material, BMJ does not warrant the accuracy and reliability of the translations (including but not limited to local regulations, clinical guidelines, terminology, drug names and drug dosages), and is not responsible for any error and/or omissions arising from translation and adaptation or otherwise.

Open access This is an open access article distributed in accordance with the Creative Commons Attribution 4.0 Unported (CC BY 4.0) license, which permits others to copy, redistribute, remix, transform and build upon this work for any purpose, provided the original work is properly cited, a link to the licence is given, and indication of whether changes were made. See: https://creativecommons.org/ licenses/by/4.0/.

ORCID IDs

Muhammad Ahmed https://orcid.org/0000-0002-9260-8958
Dominic Oliver https://orcid.org/0000-0002-8920-3407
Paolo Fusar-Poli https://orcid.org/0000-0003-3582-6788
Rashmi Patel http://orcid.org/0000-0002-9259-8788

For peer review only

Natural Language Processing enriched electronic health record data studies to investigate the associations of remote mental health care with clinical outcomes in psychiatry.

References

1. Patel R, Irving J, Brinn A, et al. Impact of the COVID-19 pandemic on remote mental healthcare and prescribing in psychiatry: an electronic health record study. *BMJ Open* 2021; **11**(3): e046365.
2. Stewart R, Martin E, Broadbent M. Mental health service activity during COVID-19 lockdown: South London and Maudsley data on working age community and home treatment team services and mortality from February to mid-May 2020. *medRxiv* 2020: 2020.06.13.20130419.
3. Moreno C, Wykes T, Galderisi S, et al. How mental health care should change as a consequence of the COVID-19 pandemic. *Lancet Psychiatry* 2020; **7**(9): 813-24.
4. Aref-Adib G, Hassiotis A. Frontline 2020: the new age for telemental health. *Lancet Psychiatry* 2021; **8**(1): 3-4.
5. Johnson S, Dalton-Locke C, Vera San Juan N, et al. Impact on mental health care and on mental health service users of the COVID-19 pandemic: a mixed methods survey of UK mental health care staff. *Soc Psychiatry Psychiatr Epidemiol* 2021; **56**(1): 25-37.
6. Perera G, Broadbent M, Callard F, et al. Cohort profile of the South London and Maudsley NHS Foundation Trust Biomedical Research Centre (SLaM BRC) Case Register: current status and recent enhancement of an Electronic Mental Health Record-derived data resource. *BMJ Open* 2016; **6**(3): e008721.
7. Patel R, Lloyd T, Jackson R, et al. Mood instability is a common feature of mental health disorders and is associated with poor clinical outcomes. *BMJ Open* 2015; **5**(5): e007504.
8. Patel R, Jayatilleke N, Broadbent M, et al. Negative symptoms in schizophrenia: a study in a large clinical sample of patients using a novel automated method. *BMJ Open* 2015; **5**(9): e007619.
9. Patel R, Wilson R, Jackson R, et al. Cannabis use and treatment resistance in first episode psychosis: a natural language processing study. *Lancet* 2015; **385 Suppl 1**: S79.
10. Patel R, Reiss P, Shetty H, et al. Do antidepressants increase the risk of mania and bipolar disorder in people with depression? A retrospective electronic case register cohort study. *BMJ Open* 2015; **5**(12): e008341.
11. Patel R, Chesney E, Taylor M, Taylor D, McGuire P. Is paliperidone palmitate more effective than other long-acting injectable antipsychotics? *Psychol Med* 2018; **48**(10): 1616-23.
12. Stewart R, Soremekun M, Perera G, et al. The South London and Maudsley NHS Foundation Trust Biomedical Research Centre (SLAM BRC) case register: development and descriptive data. *BMC Psychiatry* 2009; **9**: 51.
13. Fernandes AC, Cloete D, Broadbent MT, et al. Development and evaluation of a de-identification procedure for a case register sourced from mental health electronic records. *BMC Med Inform Decis Mak* 2013; **13**: 71.
14. Jackson MRG, Ball M, Patel R, Hayes RD, Dobson RJ, Stewart R. TextHunter--A User Friendly Tool for Extracting Generic Concepts from Free Text in Clinical Research. *AMIA Annu Symp Proc* 2014; **2014**: 729-38.
15. Maudsley Biomedical Research Centre. Library of production-ready applications. 2020. https://www.maudsleybrc.nihr.ac.uk/media/325736/applications-library-v13.pdf (accessed June 23, 2022.
16. Ghebreyesus TA. WHO Director-General's opening remarks at the media briefing on COVID-19 - 11 March 2020,. 2020. https://www.who.int/dg/speeches/detail/who-director-general-s- 38 opening-remarks-at-the-media-briefing-on-covid-19-11-march- 2020

For peer review only - http://bmjopen.bmj.com/site/about/guidelines.xhtml

17. Maudsley Biomedical Research Centre. Clinical Record Interactive Search (CRIS). 2022. https://www.maudsleybrc.nihr.ac.uk/facilities/clinical-record-interactive-search-cris/ (accessed 12 July, 2022.
18. Greater London Authority. Indices of Deprivation. 2020. https://data.london.gov.uk/dataset/indices-of-deprivation) (accessed June 23, 2022.
19. National Health Service. NHS Data Model and Dictionary. 2022. https://www.datadictionary.nhs.ukJune 23, 2022).
20. Jackson RG, Patel R, Jayatilleke N, et al. Natural language processing to extract symptoms of severe mental illness from clinical text: the Clinical Record Interactive Search Comprehensive Data Extraction (CRIS-CODE) project. *BMJ Open* 2017; **7**(1): e012012.
21. Patel R, Wilson R, Jackson R, et al. Association of cannabis use with hospital admission and antipsychotic treatment failure in first episode psychosis: an observational study. *BMJ Open* 2016; **6**(3): e009888.
22. Nuzum E, Martin E, Broadbent M, Stewart R. Mental health service activity during COVID-19 lockdown among individuals with Personality Disorders: South London and Maudsley data on services and mortality from January to May 2020. *medRxiv* 2020: 2020.09.13.20193730.
23. Santesteban-Echarri O, Piskulic D, Nyman RK, Addington J. Telehealth interventions for schizophrenia-spectrum disorders and clinical high-risk for psychosis individuals: A scoping review. *J Telemed Telecare* 2020; **26**(1-2): 14-20.
24. Zaylor C. Clinical outcomes in telepsychiatry. *J Telemed Telecare* 1999; **5 Suppl 1**: S59-60.
25. Eze ND, Mateus C, Cravo Oliveira Hashiguchi T. Telemedicine in the OECD: An umbrella review of clinical and cost-effectiveness, patient experience and implementation. *PLoS One* 2020; **15**(8): e0237585.
26. Sheehan R, Dalton-Locke C, Ali A, Totsika V, Vera San Juan N, Hassiotis A. Mental healthcare and service user impact of the COVID-19 pandemic: results of a UK survey of staff working with people with intellectual disability and developmental disorders. *medRxiv* 2020: 2020.09.01.20178848.
27. Hilty DM, Ferrer DC, Parish MB, Johnston B, Callahan EJ, Yellowlees PM. The effectiveness of telemental health: a 2013 review. *Telemed J E Health* 2013; **19**(6): 444-54.
28. Hyler SE, Gangure DP, Batchelder ST. Can telepsychiatry replace in-person psychiatric assessments? A review and meta-analysis of comparison studies. *CNS Spectr* 2005; **10**(5): 403-13.
29. Goldberg SB, Buck B, Raphaely S, Fortney JC. Measuring Psychiatric Symptoms Remotely: a Systematic Review of Remote Measurement-Based Care. *Curr Psychiatry Rep* 2018; **20**(10): 81.
30. Sharp IR, Kobak KA, Osman DA. The use of videoconferencing with patients with psychosis: a review of the literature. *Ann Gen Psychiatry* 2011; **10**(1): 14.

For peer review only - http://bmjopen.bmj.com/site/about/guidelines.xhtml

Natural Language Processing enriched electronic health record data studies to investigate the associations of remote mental health care with clinical outcomes in psychiatry.

Online Supplementary Material

Supplementary Table 1 Natural language processing applications

Application	Description	Example	Precision and recall
Symptoms and signs			
Aggression	Application to identify instances of aggressive behaviour in patients, including verbal, physical and sexual aggression.	'Reported to be quite aggressive'	P=91% R=75%
Agitation	Application to identify instances of agitation.	'Very agitated at present'	P=85% R=79%
Anergia	Application to identify instances of anergia.	'Feelings of anergia'	P=95% R=89%
Anhedonia	Application to identify instances of anhedonia (inability to experience pleasure from activities usually found enjoyable).	'No evidence of anhedonia'	P=93% R=86%
Apathy	Application to extract the presence of apathy.	'Continues to demonstrate apathy'	P=93% R=86%
Arousal	Application to identify instances of arousal excluding sexual arousal.	'Hyperaroused state"	P=89% R=91%
Bad Dreams	Application to identify instances of experiencing a bad dream.	'Frequently has bad dreams	P=89% R=100%
Blunted Affect	Application to identify instances of blunted affect.	'Affect remains very blunted'	P=100% R=80%
Circumstantiality	Application to identify instances of circumstantiality.	'Loose associations and circumstantiality'	P=94% R=92%
Concrete Thinking	Application to identify instances of concrete thinking.	'No evidence of concrete thinking'	P=84% R=41%
Delusions	Application to identify instances of delusions.	'Paranoid delusions'	P=93% R=85%
Derailment	Application to identify instances of derailment.	'He derailed frequently'	P=84% R=99%
Disturbed Sleep	Application to identify instances of disturbed sleep.	'Complains of poor sleep'	P=88% R=68%
Drowsiness	Application to identify instances of drowsiness.	'She has complained of feeling drowsy'	P= 80% R=100%
Echolalia	Application to extract occurrences where echolalia is present.	'Intermittent echolalia'	P=89% R=86%
Elation	Application to identify instances of elation.	'Mildly elated in mood'	P=94% R=97%

Emotional Withdrawal	Application to identify instances of emotional withdrawal.	'Patient withdrawn'	P=85% R=96%
Flight of Ideas	Application to extract instances of flight of ideas.	'Some flight of ideas.	P=91% R=94%
Formal Thought Disorder	Application to extract occurrences where formal thought disorder is present.	'Deteriorating into a more thought disordered state'	P=83% R=83%
Grandiosity	Application to extract occurrences where grandiosity is apparent.	'Reduction in grandiosity'	P=95% R=91
Guilt	Application to identify instances of guilt.	'No longer feels guilty'	P=83% R=83%
Hallucinations (All**)**	Application to identify instances of hallucinations.	'Doesn't appear distressed by his hallucinations'	P=84% R=98%
Hallucinations (Auditory)	Application to identify instances of auditory hallucinations non-specific to diagnosis.	'Experiencing auditory hallucinations'	P= 80%, R=84%
Hallucinations (Olfactory/Tactile/Gustatory)	Application to extract occurrences where hallucination ((Olfactory/Tactile/Gustatory) is present.	'Seems to be having olfactory hallucinations, in relation to her tactile hallucinations'	P=78%, R=68%
Hallucinations (Visual)	Application to extract occurrences where visual hallucination is present. Visual hallucinations may be due to a diagnosis of psychosis/schizophrenia or may be due to other causes, e.g., due to substance abuse.	'Experiencing visual hallucination'	P=91%, R=96%
Helplessness	Application to identify instances of helplessness.	'Ideas of helplessness'	P=93% R=86%
Hopelessness	Application to identify instances of hopelessness.	'Says feels hopeless'	P=90% R=95%
Hostility	Application to identify instances of hostility.	'Increased hostility and paranoia'	P=89%, R=94%
Insomnia	Application to identify instances of insomnia.	'Complaining of insomnia'	P=89%, R=94%
Irritability	Application to identify instances of irritability.	'Became irritable'	P=100% R=83%
Loss of Coherence	Application to identify instances of incoherence or loss of coherence in speech or thinking.	'Patient was incoherent'	P=98% R=95%
Low Energy	Application to identify instances of low energy.	'Decreased energy'	P=72% R=67%
Mood Instability	This application identifies instances of mood instability.	'Expressed fluctuating mood'	P=100% R=70%
Mutism	Application to identify instances of mutism.	'Did not respond any further and remained mute'	P=91% R=75%

For peer review only

Negative Symptoms	Application to identify instances of negative symptoms.	'Diagnosis of schizophrenia with prominent negative symptoms'	P=86% P=95%
Nightmares	Application to identify instances of nightmares.	'Unsettled sleep with vivid nightmares'	P=89% R=100%
Paranoia	Application to identify instances of paranoia. Paranoia may be due to a diagnosis of paranoid schizophrenia or may be due to other causes, e.g., substance abuse.	'Vague paranoid ideation'	P=86%, R=94%
Passivity	Application to identify instances of passivity.	'Patient describes experiencing passivity'	P=89% P=100%
Persecutory Ideation	Application to identify instances of ideas of persecution.	'Marked persecutory delusions'	P=80% R=96%
Poor Appetite	Application to identify instances of poor appetite (negative annotations).	'Normal appetite'	P=83% R=71%
Poor Concentration	Application to identify instances of poor concentration.	'Finds it hard to concentrate'	P=84% R=60%
Poor Insight	Applications to identify instances of poor insight.	'Limited insight'	P=87% R=70%
Poor Motivation	This application aims to identify instances of poor motivation.	'Struggling with motivation'	P=95% R=38%
Poverty of Speech	Application to identify poverty of speech.	'Continues to display negative symptoms including poverty of speech'	P=87% R=85%
Poverty of Thought	Application to identify instances of poverty of thought.	'Evidence of poverty of thought'	P=95%, R=93%
Social Withdrawal	Application to identify instances of social withdrawal.	'Very isolated and socially withdrawn'	P=60% R=86%
Stupor	Application to identify instances of stupor. This includes depressive stupor, psychotic stupor, catatonic stupor, dissociative stupor and manic stupor.	'Man with stuporous catatonia'	P=88% R=87%
Suicidal Ideation	Application to identify instances of suicidal ideation - thinking about, considering, or planning suicide.	'No suicidal ideation disclosed'	P=81% R=87%
Tangentiality	Application to identify instances of tangentiality.	'Evidence of tangential speech'	P=99% R=90%
Tearfulness	Application to identify instances of tearfulness.	'Appeared tearful'	P=100% R=94%
Thought Block	Application to identify instances of thought block.	'Showed some thought block'	P=91% R=75%

For peer review only

Thought Broadcast	Application to identify instances of thought broadcasting.	'Patient describes experiencing thought broadcasting'	P=86% R=92%
Thought Insertion	Application to identify instances of thought insertion.	'No thought insertion'	P=81% R=96%
Thought Withdrawal	Application to identify instances of thought withdrawal.	'Presence of thought withdrawal'	P=90% R=88%
Waxy Flexibility	Application to identify instances of waxy flexibility. Waxy flexibility is a psychomotor symptom of catatonia as associated with schizophrenia, bipolar disorder, or other mental disorders which leads to a decreased response to stimuli and a tendency to remain in an immobile posture.	'Presents as catatonic with waxy flexibility'	P=80% R=86%
Weight Loss	Application to identify instances of weight loss.	'Significant weight loss'	P=90% R=88%
Worthlessness	Application to identify instances of worthlessness.	'Feeling worthless'	P=88% P=86%
Contextual factors			
Asthma	Application to identify patients with diagnosis of asthma.	'Suffered from an asthma attack'	P=95% R=84%
Bronchitis	Application to identify patients with diagnosis of bronchitis.	'Past diagnosis: chronic obstructive airway disease'	P=85% R=48%
Cough	Application to identify instances of coughing.	'Denied any coughing or shortness of breath'	P=83% R=80%
Crohn's Disease	Application to identify patients with diagnosis of Crohn's disease.	'Has Crohn's disease'	P=94% R=78%
Hypertension	Application to identify patients with diagnosis of hypertension or high blood pressure.	'History of hypertension'	P=94% R=94%
Rheumatoid Arthritis	Application to identify patients with diagnoses of rheumatoid arthritis.	'Pain due to her rheumatoid arthritis'	R=91% R=86%
Amphetamine	To identify instances of amphetamine use.	'Has not used amphetamine for the last week'	P=80% R=84%
Cannabis	To identify instances of cannabis use.	'No cannabis use'	P=77% R=93%
Cocaine/Crack Cocaine	To identify instances of cocaine or crack cocaine use.	'He has stopped taking cocaine'	P=84% R=97%
MDMA	Application to identify instances of MDMA use.	'First took MDMA at the age of 15'	P=100% R=99%
Tobacco Smoking	This application distinguishes between people who are a) current smokers, b) current	'Doesn't smoke'	P=81% R=74%

	non-smokers (ever smoked) and c) non-smokers.		
Lives Alone	Application to identify instances of living alone.	'Lives on her own'	P=77% R=83%
Domestic Violence;	Application to identify instances of domestic violence.	'Victim of DV'	P=86% R=93%
Loneliness	Application to identify instances of loneliness.	'Patient is lonely'	P= 87% R= 100%
Interventions			
Cognitive Behavioural Therapy (CBT)	An application to identify instances of delivered sessions of Cognitive Behavioural Therapy (CBT).	'Session of CBT'	P=99% R=82%
Family Intervention	The application identifies instances of family intervention delivery.	"Family therapy"	P=77% R=87%
Medication	The application identifies instances of medication prescription.	'Quetiapine'	Antipsychotics: P=97%-99%, R=39%
Outcomes and Clinical Status			
Blood Pressure (BP)	Application to identify instances of blood pressure scores in the format of overall score, systolic blood pressure score and diastolic blood pressure score.	'140/90mmHg'	P=98% R=96%
Body Mass Index (BMI)	Application to identify body mass index (BMI) scores.	'Body Mass Index is 22.9'	P=89% R=78%
HbA1c	Application to identify HbA1c results.	'HbA1c was 40mmol/mol'	P=89% R=93%
Mini-Mental State Examination (MMSE)	This app identifies MMSE scores.	'25/30'	P=93% R=94%
Diagnosis	Application to extract instances of diagnosis.	'Schizophrenia'	F20: P=100%, R=65%
Treatment-Resistant Depression	Application to identify instances of treatment-resistant depression.	'History of treatment resistant depression'	P=77% R=95%
Bradykinesia (Dementia)	To identify instances of bradykinesia in the context of dementia.	'Moderate bradykinesia'	P=91% R=84%
Tremor (Dementia)	Application to identify instances of tremor in patients with dementia.	'With a degree of resting tremor'	P=83%, R=92%

For peer review only

BMJ Open

Associations of remote mental healthcare with clinical outcomes: a natural language processing enriched electronic health record data study protocol.

Journal:	*BMJ Open*
Manuscript ID	bmjopen-2022-067254.R1
Article Type:	Protocol
Date Submitted by the Author:	12-Jan-2023
Complete List of Authors:	Ahmed, Muhammad; Institute of Psychiatry Psychology and Neuroscience Division of Academic Psychiatry, Department of Psychosis Studies<br>Kornblum, Daisy; South London and Maudsley Mental Health NHS Trust, NIHR Maudsley Biomedical Research Centre<br>Oliver, Dominic; Institute of Psychiatry Psychology and Neuroscience Division of Academic Psychiatry, Department of Psychosis Studies<br>Fusar-Poli, Paolo; Institute of Psychiatry Psychology and Neuroscience Division of Academic Psychiatry, Department of Psychosis Studies<br>Patel, Rashmi; Institute of Psychiatry Psychology and Neuroscience Division of Academic Psychiatry, Department of Psychological Medicine; South London and Maudsley Mental Health NHS Trust, NIHR Maudsley Biomedical Research Centre
**Primary Subject Heading**:	Mental health
Secondary Subject Heading:	Epidemiology, Evidence based practice, Health informatics
Keywords:	EPIDEMIOLOGY, Telemedicine < BIOTECHNOLOGY & BIOINFORMATICS, Health informatics < BIOTECHNOLOGY & BIOINFORMATICS, PSYCHIATRY

SCHOLARONE™
Manuscripts

Associations of remote mental healthcare with clinical outcomes: a natural language processing enriched electronic health record data study protocol

Muhammad Ahmed[1*], Daisy Kornblum[2], Dominic Oliver[1], Paolo Fusar-Poli[1,2], Rashmi Patel[2,3*]

[1]Department of Psychosis Studies, King's College London, Institute of Psychiatry Psychology and Neuroscience, London, UK

[2] NIHR Maudsley Biomedical Research Centre, South London and Maudsley NHS Foundation Trust, London, UK

[3]Department of Psychological Medicine, King's College London, Institute of Psychiatry Psychology and Neuroscience, London, UK

*Equal contribution to authorship

Correspondence to:

Rashmi Patel

bmj@rpatel.co.uk

Word count: 3,530

Keywords: natural language processing, electronic health records, remote mental healthcare

For peer review only

Associations of remote mental healthcare with clinical outcomes: a natural language processing enriched electronic health record data study protocol.

Abstract

Introduction People often experience significant difficulties in receiving mental healthcare due to insufficient resources, stigma and lack of access to care. Remote care technology has the potential to overcome these barriers by reducing travel time and increasing frequency of contact with patients. However, the safe delivery of remote mental healthcare requires evidence on which aspects of care are suitable for remote delivery and which are better served by in-person care. We aim to investigate clinical and demographic associations with remote mental healthcare in a large electronic health record (EHR) dataset and the degree to which remote care is associated with differences in clinical outcomes using natural language processing (NLP) derived EHR data.

Methods and analysis De-identified EHR data, derived from the South London and Maudsley (SLaM) NHS Foundation Trust Biomedical Research Centre (BRC) Case Register, will be extracted using the Clinical Record Interactive Search tool for all patients receiving mental healthcare between 1st January 2019 and 31st March 2022. First, data on a retrospective, longitudinal cohort of around 80,000 patients will be analysed using descriptive statistics to investigate clinical and demographic associations with remote mental healthcare and multivariable Cox regression to compare clinical outcomes of remote vs in-person assessments. Second, NLP models that have been previously developed to extract mental health symptom data will be applied to around 5 million documents to analyse the variation in content of remote vs in-person assessments.

Ethics and dissemination The SLaM BRC Case Register and CRIS tool have received ethical approval as a de-identified dataset (including NLP-derived data from unstructured free text documents) for secondary mental health research from Oxfordshire REC C (Ref: 18/SC/0372). The study has received approval from the SLaM CRIS Oversight Committee. Study findings will be disseminated through peer-reviewed, open access journal articles and service user and carer advisory groups.

For peer review only - http://bmjopen.bmj.com/site/about/guidelines.xhtml

Associations of remote mental healthcare with clinical outcomes: a natural language processing enriched electronic health record data study protocol.

Article summary

Strengths and limitations of this study

- The use of electronic health record data will enable comparisons between remote vs in-person assessments in a large sample of NHS patients with mental health disorders which is representative of real-world clinical practice.
- Natural language processing models will enable analysis of free text electronic health record data to ascertain differences in clinical presentation, assessment and outcomes between patients receiving remote and in-person assessments.
- Routinely recorded data will be investigated for secondary research purposes. However, data on clinical outcomes may be missing or inaccurate. Data availability relies on the recording of accurate data at the point of clinical contact.
- As this is an observational study, associations with remote mental healthcare could be confounded by indication and it will not be possible to infer any causality between observed associations with clinical outcomes.
- The study will analyse secondary mental health data which does not include data from primary care.

For peer review only

Associations of remote mental healthcare with clinical outcomes: a natural language processing enriched electronic health record data study protocol.

Introduction

Remote technologies such as phone, video calls, text messages and e-mail allow patients and clinicians to communicate with each other when it is not possible to meet in-person. The COVID-19 pandemic accelerated the implementation of remote care technology in mental health with services often using remote technology to maintain clinical contact and deliver care[1 2]. Although restrictions resulting from the pandemic have eased, remote technology is likely to continue to be used and remain a useful adjunct to conventional in-person consultation[3].

Access to conventional mental healthcare may be hindered due to lack of resources, stigma, and lack of access to community mental health services[1]. Remote care technology has the potential to overcome these barriers and augment in-person care[4]. Additionally, remote technology has proved surprisingly popular with both clinicians and patients[3]. However, some patients are likely to find remote technology less helpful than others. They may lack necessary equipment and reliable internet connectivity, be unfamiliar and uncomfortable with the technology, or have mental disorders such as dementia[5] and schizophrenia[6] that affect their motivation and cognitive functioning making it difficult to participate in remote care. Patients, carers and clinicians may also have concerns about privacy and security[7]. The potential value of remote technology in mental healthcare has yet to be systematically evaluated and the evidence base to support best practice is sparse. The potential benefits of remote care must be balanced against the risk that its use might, in some situations, have adverse effects on quality of care, patient satisfaction, and clinical outcomes. There is a pressing need to formally evaluate the impact of remote care technology on mental healthcare so that potential barriers to accessing remote care can be identified, its benefits maximised, and potential risks minimised.

Electronic health record (EHR) data could be used to address this gap in understanding. EHRs have been widely adopted in NHS healthcare services[8]. Information is stored as both structured data, which includes categorical data such as demographics, prescribed medications, and diagnoses, and, to a greater extent, unstructured data such as free text notes which tend to be written in narrative format by clinical or administrative staff. Unstructured data may contain details on symptoms, diagnoses, and treatment plans that are otherwise not available in structured data fields. EHRs not only support individual patient care but also have the potential to support largescale research through the analysis of de-identified clinical data[9]. Natural language processing (NLP) techniques can be used to automatically and rapidly extract clinical information from unstructured free text clinical notes and correspondence to assess the associations of clinical factors,[9 10] environmental factors[11] and treatments[12 13] with clinical outcomes.[14]

We aim to investigate the prevalence and distribution of remote assessments to better understand variation in uptake based on clinical and demographic characteristics and to investigate differences in clinical outcomes associated with remote vs in-person assessments using a large sample of patients receiving care for mental disorders by applying NLP to extract data from de-identified EHR records.

The rationale for conducting this study is to contribute to the evidence base related to the use of remote mental healthcare that can positively impact clinical practice and service delivery. The ability to support greater access to mental healthcare through remote technology would reduce barriers and costs in providing care to people who would benefit from this approach while increasing capacity to provide in-person care to those who would prefer or cannot be safely treated remotely. This could enable more patients to receive timely care and improve clinical outcomes, quality of life and functioning as improve cost-effectiveness of mental healthcare provision.

For peer review only

Methods and Analysis

Study aims and design

We will conduct two studies. First, we aim to conduct a retrospective, longitudinal cohort study to examine the potential associations between remote care and clinical outcomes and to test the hypothesis that (a) use of remote care will be lower in patients with poor motivation compared to in-person care, and (b) remote care use will be more frequent in the treatment of less severe disorders, such as mild depression and anxiety, whereas in-person care will be more frequent in the treatment of more severe disorders, such as psychotic and bipolar mood disorders.

Second, an NLP study which will compare clinical documentation between in-person appointments and remote care appointments. We hypothesise that (a) the documentation of diagnosis, symptoms and treatments will be similar for remote and in-person care, (b) documentation of clinical signs (as part of mental state examination) will be less frequent with remote than with in-person care, and (c) use of remote care will be associated with fewer documented episodes of suicidal thoughts. We hypothesise that differences between remote and in-person care will be less evident when remote assessment involves video, as opposed to audio or text.

Study setting

The data used in this study will be obtained from the South London and Maudsley (SLaM) National Health Service (NHS) Foundation Trust. SLaM is one of the largest providers of specialist mental healthcare in Europe and provides more than 230 services, including specialist psychosis services, inpatient wards, community and outpatient services, within its catchment serving approximately 1.3 million residents in the London boroughs of Lambeth, Southwark, Lewisham and Croydon. SLaM treats over 5,000 inpatients and 40,000 outpatients per year. The Trust has implemented a fully electronic health record system since 1 April 2006 and the SLaM NIHR Biomedical Research Centre (BRC) Case Register contains the clinical records of 417,482 patients (accurate on 08/02/2022)[15].

Data source

Since April 2006, SLaM has utilised an electronic psychiatric clinical records system, the electronic Patient Journey System (ePJS) to record instances of patients accessing or interacting with mental healthcare services. The SLaM BRC Case Register comprises de-identified clinical data from ePJS including structured fields (for demographic, diagnostic and medication data) and de-identified unstructured free text fields from case notes and correspondence. Unstructured clinical text is documented by healthcare professionals during the provision of mental healthcare to patients and includes history, mental state examination, diagnostic formulation and care plans. Healthcare professionals who document clinical data include psychiatrists, psychologists, nursing staff, care coordinators and allied healthcare professionals. A patient-led oversight committee considers all proposed research before access to the anonymised data is permitted and access to data is restricted to honorary or substantive employees of SLaM[16].

Data for this study will be obtained from the SLaM BRC Case Register using the Clinical Record Interactive Search tool (CRIS), a bespoke Microsoft Structured Query Language (SQL) database query and assembly tool that allows authorised researchers to extract and recode data from the SLaM BRC Case Register. Data are obtained from structured electronic health record fields and from unstructured free-text documentation using natural language processing (NLP)[8]. Data analysis will commence on 1st October 2022.

For peer review only - http://bmjopen.bmj.com/site/about/guidelines.xhtml

Natural language processing

NLP is an information extraction technique used to automatically identify relevant information from unstructured free text. NLP models are developed by defining a construct of interest (e.g. a particular clinical symptom or outcome), assembling a corpus of documents potentially containing the construct of interest (e.g. a selection of de-identified clinical documents from EHRs) and manually annotating examples from the corpus to classify true positive instances of the construct of interest. The annotated examples may be used as training and reference data to develop and evaluate the accuracy of a machine learning-derived NLP algorithm to automatically identify the presence of the construct in other documents.

To maximise ascertainment of clinical data in this study, as well as assembling structured data, NLP models which have previously been developed to extract mental disorder symptom data from EHR documents will be applied.[17] These NLP models have been developed to enrich the SLaM BRC Case Register by allowing clinical information to be extracted from unstructured free text which comprise assessments, reviews and correspondence documented by mental healthcare professionals. The data extracted by NLP include a range of symptoms, contextual factors (certain physical health disorders, exposure to illicit substances and social factors), interventions (medications and psychological therapy), and clinical outcomes.[17,18]

The performance of an NLP model is measured through precision and recall. Precision refers to the number of true positive instances that the NLP model has identified, divided by the total number of instances retrieved by the NLP model (both true positive and false positive instances). Recall refers to the number of true positive instances identified by the NLP model, divided by the total number of existing true positives in the entire sample. Precision and recall statistics for the NLP models employed in this project are supplied in supplementary table 1. The full library of NLP models available within CRIS and details of their development are provided on the SLaM CRIS website[18].

NLP-derived data will be ascertained as binary variables which indicate the presence of at least one mention of the construct of interest in each analysed document.

Study population

Data will be extracted for all patients of all ages who had an active episode of care from 1st January 2019 until 31st March 2022. An active episode of care is defined as at least one clinical contact with a healthcare professional with SLaM services during the study period.

Index dates

Data extracted for both studies will be from the first active episode of care between 1st January 2019 to 31st March 2022. This time-period was selected to provide data on clinical assessments prior to and during the COVID-19 pandemic (declared by the World Health Organisation in March 2020)[19], and on follow-up data relating to longitudinal clinical outcomes.

The index date for both studies will be the 1st of January 2019. For the longitudinal cohort study, all patients who have an active episode of care during 2019 and during 2020 will be considered. Follow-up data will be obtained from 1st January 2021 to 31st March 2022. For the NLP study, data on clinical assessments and clinical outcomes will be considered from 1st January 2019 to 31st March 2022.

Data extraction

Data derived from the SLaM BRC Case register will be interrogated using Structured Query Language (SQL), a standardised language for accessing and manipulating relational databases. SQL

For peer review only - http://bmjopen.bmj.com/site/about/guidelines.xhtml

enables the application of queries to gather data from different parts of the SLaM BRC Case Register at the individual patient level. The use of SQL with electronic health record databases allows researchers to identify specific patient samples and extract relevant longitudinal clinical data. Demographic data on age, gender, ethnicity, marital status, occupation, housing status, multiple deprivation score and date of death, and clinical data on psychiatric disorder diagnosis and comorbid diagnoses will be extracted using SQL.

Data on type of clinical event attended (in-person vs remote including did not attend), type of remote event (video, audio or text), number of hospital admissions (sections and voluntary), date of hospital admission, date of discharge, number of A&E Liaison Psychiatry care episodes, number of Crisis Resolution Home Treatment Team (CRHTT) care episodes and whether psychotropic medications (antidepressants, antipsychotics and mood stabilisers) were prescribed will be extracted to support descriptive and inferential analyses.

Patient and public involvement

The study was developed in collaboration with the SLaM CRIS Service User and Carer Advisory Group (SUCAG), which includes patients and carers who have experience of SLaM mental healthcare and experience of supporting EHR data research studies. The study has received approval from the SLaM CRIS Oversight Committee (chaired by a patient representative) which reviews and approves applications to extract and analyse the data. The CRIS infrastructure was designed and is managed with ongoing patient and carer input to ensure all research projects comply with data governance, legal and ethical guidelines. Further details are available on the SLaM CRIS website[20].

Defined variables

The predictor variables and covariates for both the Cohort and NLP studies will be the same.

Predictors

The type of clinical event attended by a patient, categorised as in-person, remote (video call, phone, letter, instant or short message service (SMS) text message, e-mail), or did not attend (DNA). Inpatient clinical events will be excluded (as they are conducted in-person) and only data from community clinical events will be analysed. A composite predictor variable will be generated as the proportion of all appointments (denominator) that are remote care appointments (numerator) between 1st January 2019 until 31st March 2022 as a measure of exposure to remote care. Also, a variable indicating the presence of documented poor motivation will be generated from unstructured text using NLP. To take into account potential differences in the use of remote care during periods of travel restrictions during the COVID-19 pandemic, supplementary analyses will be conducted for the period between 1st March 2020 and 31st July 2021.

Covariates

Age, gender, ethnicity, occupation, marital status, psychiatric disorder ICD-10 diagnosis (categorised according to major disorder groups defined in a previous study[21]), housing status, and index of social deprivation will be obtained through a data linkage with the London Data Store[22] at Lower Super Output Area (LSOA) level. For patients in the cohort with at least one clinical event documented on or prior to 31st December 2017, a supplementary analysis will be conducted (provided a sufficient sample size is available) in which the number of appointments and number of inpatient admissions during 2018 will be included as a covariate to adjust for the potential association of prior healthcare service utilisation with clinical outcomes.

For peer review only - http://bmjopen.bmj.com/site/about/guidelines.xhtml

Covariate categories will be coded according to NHS Data Dictionary definitions[23]. All predictor and covariate variables will be obtained from structured EHR data fields except for psychiatric disorder diagnosis, marital status and housing status which will be obtained from both structured and unstructured data using NLP using methods described subsequently.

Outcome measures

Cohort study

Primary outcome variable: psychiatric hospital admissions categorised as voluntary or involuntary (under a section of the UK Mental Health Act). Psychiatric hospital admission was chosen as the primary outcome variable to compare illness severity associated with the type of clinical event attended by a patient.

Secondary outcome variables: A&E Liaison Psychiatry care episodes, Crisis Resolution Home Treatment Team (CRHTT) care episodes, psychotropic medication prescribing (antidepressants, antipsychotics and mood stabilisers defined according to the British National Formulary[24]), ICD-10 psychiatric disorder diagnosis, attendance/non-attendance of in-person appointments, attendance/non-attendance of remote care appointments, and mortality as recorded in EHR data and through a pre-existing data linkage with the Office for National Statistics (ONS) to obtain data on cause of death including natural causes, suicide or open coroners' verdict, or other unnatural causes (unlawful killing, accident or misadventure).

All outcome variables will be obtained from structured EHR data fields.

NLP Study

Outcome variable: difference in frequency in documentation of diagnosis, symptoms, clinical signs (as part of mental state examination), treatments and episodes of suicidal thoughts between clinical documents related to in-person appointments compared to documents related to remote care appointments.

Statistical analysis

Data analysis will be performed using R statistical computing and graphics software.

Cohort study

1. Descriptive statistics

Descriptive statistics will be obtained for demographic variables, including age, gender, ethnicity, marital status, housing status, occupation and multiple deprivation score. Frequencies and percentages will be obtained for categorical variables and mean and standard deviations will be obtained for continuous variables.

2. Multivariable logistic regression analyses

Multivariable logistic regression will be used to analyse the potential association between type of clinical event attended and (i) psychiatric hospital admission; (ii) A&E Liaison Psychiatry care episodes; (iii) Crisis Resolution Home Treatment Team (CRHTT) care episodes; (iv) primary

For peer review only - http://bmjopen.bmj.com/site/about/guidelines.xhtml

psychiatric diagnosis; (v) comorbid diagnosis; (vi) antidepressant, antipsychotic and mood stabiliser prescription and (vii) mortality.

Multivariable logistic regression will be used to analyse the potential association between (i) poor motivation and clinical event attendance; (ii) primary psychiatric disorder diagnosis and clinical event attendance; and (iii) comorbid psychiatric disorder diagnosis and clinical event attendance.

3. Multivariable cox regression analyses

Multivariable Cox regression will be used to analyse the potential association between type of clinical event attended and time to (i) voluntary psychiatric hospital admission; (ii) involuntary psychiatric hospital admission; (iii) A&E Liaison Psychiatry care episodes; (iv) Crisis Resolution Home Treatment Team (CRHTT) care episodes; (v) primary psychiatric disorder diagnosis; (vi) comorbid psychiatric disorder diagnosis; (vii) antidepressant, antipsychotic and mood stabiliser prescription and (viii) mortality.

NLP Study

NLP will be used to obtain data using applications previously developed by RP in collaboration with the NIHR Maudsley Biomedical Research Centre (BRC)[9 10 25 26]. Supplementary table 1 summarises the NLP applications and full details of the specification and precision statistics are available from the CRIS NLP Library[18].

NLP data will be obtained for each unstructured document representing an in-person or remote care event. The number of in-person vs remote care documents containing each NLP-derived construct will be compared using Chi-squared tests and logistic regression (predictor variable: type of clinical contact; outcome variable: presence of NLP-derived construct).

Sample size

As sample sizes of number of patients and number of documents in the SLaM BRC Case Register can be estimated from previous years' data, the pwr package (R v4.0.2) was used to estimate the minimal detectable effect size (MDE) for the cohort study and NLP study analyses using a significance level of 0.001 and power of 0.8 as follows:

Cohort study: MDE Cohen's w (sample size: 78,805 active patients in 2018 and 2019; df: 20): 0.022

NLP Study: MDE Cohen's w (sample size: 5,083,863 documents in 2018 and 2019; df: 100): 0.004

This indicates the data available will be well powered to detect small changes in effect size.

Missing data

Based on previous studies, data on the predictor variable, age, gender and all outcome variables will be complete within the SLaM BRC Case Register. Where there are missing data for other covariates, two analyses will be conducted: a full sample analysis including missing data as a category in regression analyses and a complete case analysis dropping individuals who have one or more missing covariates.

For peer review only - http://bmjopen.bmj.com/site/about/guidelines.xhtml

Ethics and dissemination

The SLaM BRC Case Register and CRIS tool have received ethical approval as a de-identified dataset for secondary mental health research analyses from Oxfordshire REC C (Ref: 18/SC/0372). The study has received approval from the SLaM CRIS Oversight Committee (chaired by a patient representative) which reviews and approves applications to extract and analyse the data. The CRIS infrastructure was designed and is managed with ongoing patient and carer input to ensure all research projects comply with data governance, legal and ethical guidelines. Further details are available on the SLaM CRIS website[20]. Study findings will be disseminated through peer-reviewed journals and published open access to ensure there are no limitations to readership and access to their contents are as widely available as possible.

Discussion

Strengths

This study aims to investigate the associations of remote mental healthcare with clinical outcomes by analysing structured and unstructured data from a large, de-identified EHR dataset. The study is strengthened by the use of routinely recorded data that are representative of clinical practice. Thus, the findings of the study will be generalisable to patients receiving care for a wide range of mental disorders and across a broad range of sociodemographic characteristics. The study will provide key insights into the factors associated with higher or lower rates of remote care appointments and provide insights for healthcare policymakers to reduce barriers to access remote care and contribute to evidence-based guidelines for best practice.

The availability of unstructured EHR data will enable the assembly and analysis of rich clinical symptom data through the use of NLP to better understand how remote care appointments vary from in-person appointments in terms of the nature of clinical information documented by mental healthcare professionals. This information could help better understand the potential benefits and limitations of remote care based on clinical presentation and treatment needs.

Limitations

As this study is observational and based on data that have already been recorded as part of routine healthcare delivery, it will not be possible to determine or infer causal associations between exposure to remote care and clinical outcomes. This would require a prospectively conducted study. It is also possible that our findings could be confounded by clinical indication. For example, clinicians may perceive a patient with poor motivation to be less likely to engage with remote care and may preferentially offer in-person appointments to such individuals.

The dataset analysed in the study will comprise EHR data from secondary mental healthcare services. Data from primary care services will not be available. This means that it will not be possible to draw inferences on the use of remote care for mental disorders in primary care. Furthermore, data will only be available for patients receiving care from the SLaM NHS Trust. It will not be possible to ascertain predictor, covariate or clinical outcome data for patients who are discharged during the period of the study (e.g. due to moving outside of the SLaM catchment area).

A further limitation is the presence of missing data. As EHR data are recorded as part of routine clinical practice, data are not systematically ascertained on every potential clinical symptom or feature a patient may or may not present with. This means that there may be missing symptom data which are not documented by clinicians and so would not be ascertained by NLP models. Nonetheless, it is likely that a clinically relevant or significant feature of a patient's presentation would be documented

For peer review only

For peer review only - http://bmjopen.bmj.com/site/about/guidelines.xhtml

in the EHR if it contributes towards the nature and/or degree of the mental disorder, and/or decisions related to treatment planning.

For peer review only

Data management and confidentiality

To comply with all necessary data protection regulations, EHR data will be stored and analysed using encrypted KCL/SLaM workstations and virtual machines hosted in a UK-based Microsoft Azure datacentre.

Twitter: Rashmi Patel @RPatelDr

Contributors: The studies were conceived and designed by RP. Protocol preparation, literature review, statistical analysis plan and manuscript preparation was performed by MA, DK, DO, PFP and RP. All authors approved the final manuscript.

Funding: RP has received funding from an NIHR Advanced Fellowship (NIHR301690), a Medical Research Council (MRC) Health Data Research UK Fellowship (MR/S003118/1) and an Academy of Medical Sciences Starter Grant for Clinical Lecturers (SGL015/1020) supported by the Wellcome Trust, MRC, British Heart Foundation, Arthritis Research UK, the Royal College of Physicians and Diabetes UK.

Disclaimer: This project is funded by the National Institute for Health Research (Advanced Fellowship), grant number NIHR301690. The views expressed are those of the authors and not necessarily those of the NHS, the NIHR or the Department of Health. The funders had no role in the design and conduct of the study; collection, management, analysis and interpretation of the data; preparation, review or approval of the manuscript and decision to submit the manuscript for publication.

Competing interests: All authors have completed the ICMJE uniform disclosure form at www.icmje.org/coi_disclosure.pdf and declare: RP has received funding from Janssen and Holmusk, outside the submitted work. The other authors declare no competing interests.

Patient and public involvement: The study was developed in collaboration with the SLaM CRIS Service User and Carer Advisory Group (SUCAG), which includes patients and carers who have experience of SLaM mental healthcare and experience of supporting EHR data research studies. The study has received approval from the SLaM CRIS Oversight Committee (chaired by a patient representative) which reviews and approves applications to extract and analyse the data. The CRIS infrastructure was designed and is managed with ongoing patient and carer input to ensure all research projects comply with data governance, legal and ethical guidelines. Further details are available on the SLaM CRIS website[20].

Patient consent for publication: Not applicable.

Ethics approval: This study involves data from human participants and was approved by Oxfordshire REC C (Ref: 18/SC/0372). The CRIS data resource received ethical approval as a deidentified dataset for secondary mental health research analyses from Oxfordshire REC C (Ref: 18/SC/0372). The study was approved by the SLaM BRC CRIS oversight committee. Consent is not required to analyse the deidentified dataset for approved research studies. Patients may opt-out of inclusion in the deidentified dataset.

Provenance and peer review: Not commissioned; externally peer reviewed.

Data availability statement: No additional data are available.

Supplemental material: This content has been supplied by the author(s). It has not been vetted by BMJ Publishing Group Limited (BMJ) and may not have been peer-reviewed. Any opinions or recommendations discussed are solely those of the author(s) and are not endorsed by BMJ. BMJ

For peer review only - http://bmjopen.bmj.com/site/about/guidelines.xhtml

disclaims all liability and responsibility arising from any reliance placed on the content. Where the content includes any translated material, BMJ does not warrant the accuracy and reliability of the translations (including but not limited to local regulations, clinical guidelines, terminology, drug names and drug dosages), and is not responsible for any error and/or omissions arising from translation and adaptation or otherwise.

Open access: This is an open access article distributed in accordance with the Creative Commons Attribution 4.0 Unported (CC BY 4.0) license, which permits others to copy, redistribute, remix, transform and build upon this work for any purpose, provided the original work is properly cited, a link to the licence is given, and indication of whether changes were made. See: https://creativecommons.org/ licenses/by/4.0/.

ORCID IDs

Muhammad Ahmed: https://orcid.org/0000-0002-9260-8958
Dominic Oliver: https://orcid.org/0000-0002-8920-3407
Paolo Fusar-Poli: https://orcid.org/0000-0003-3582-6788
Rashmi Patel: http://orcid.org/0000-0002-9259-8788

For peer review only

References

1. Patel R, Irving J, Brinn A, et al. Impact of the COVID-19 pandemic on remote mental healthcare and prescribing in psychiatry: an electronic health record study. *BMJ Open* 2021;11(3):e046365. doi: 10.1136/bmjopen-2020-046365 [published Online First: 20210330]
2. Stewart R, Martin E, Broadbent M. Mental health service activity during COVID-19 lockdown: South London and Maudsley data on working age community and home treatment team services and mortality from February to mid-May 2020. *medRxiv* 2020:2020.06.13.20130419. doi: 10.1101/2020.06.13.20130419
3. Moreno C, Wykes T, Galderisi S, et al. How mental health care should change as a consequence of the COVID-19 pandemic. *Lancet Psychiatry* 2020;7(9):813-24. doi: 10.1016/s2215-0366(20)30307-2 [published Online First: 20200716]
4. Aref-Adib G, Hassiotis A. Frontline 2020: the new age for telemental health. *Lancet Psychiatry* 2021;8(1):3-4. doi: 10.1016/s2215-0366(20)30490-9
5. Chirico I, Giebel C, Lion K, et al. Use of technology by people with dementia and informal carers during COVID-19: A cross-country comparison. *International Journal of Geriatric Psychiatry* 2022;37(9) doi: https://doi.org/10.1002/gps.5801
6. Gard DE, Fisher M, Garrett C, et al. Motivation and its Relationship to Neurocognition, Social Cognition, and Functional Outcome in Schizophrenia. *Schizophrenia Research* 2009;115(1):74-81. doi: https://doi.org/10.1016/j.schres.2009.08.015
7. Johnson S, Dalton-Locke C, Vera San Juan N, et al. Impact on mental health care and on mental health service users of the COVID-19 pandemic: a mixed methods survey of UK mental health care staff. *Soc Psychiatry Psychiatr Epidemiol* 2021;56(1):25-37. doi: 10.1007/s00127-020-01927-4 [published Online First: 20200828]
8. Perera G, Broadbent M, Callard F, et al. Cohort profile of the South London and Maudsley NHS Foundation Trust Biomedical Research Centre (SLaM BRC) Case Register: current status and recent enhancement of an Electronic Mental Health Record-derived data resource. *BMJ Open* 2016;6(3):e008721. doi: 10.1136/bmjopen-2015-008721 [published Online First: 20160301]
9. Patel R, Lloyd T, Jackson R, et al. Mood instability is a common feature of mental health disorders and is associated with poor clinical outcomes. *BMJ Open* 2015;5(5):e007504. doi: 10.1136/bmjopen-2014-007504 [published Online First: 20150521]
10. Patel R, Jayatilleke N, Broadbent M, et al. Negative symptoms in schizophrenia: a study in a large clinical sample of patients using a novel automated method. *BMJ Open* 2015;5(9):e007619. doi: 10.1136/bmjopen-2015-007619 [published Online First: 20150907]
11. Patel R, Wilson R, Jackson R, et al. Cannabis use and treatment resistance in first episode psychosis: a natural language processing study. *Lancet* 2015;385 Suppl 1:S79. doi: 10.1016/s0140-6736(15)60394-4
12. Patel R, Reiss P, Shetty H, et al. Do antidepressants increase the risk of mania and bipolar disorder in people with depression? A retrospective electronic case register cohort study. *BMJ Open* 2015;5(12):e008341. doi: 10.1136/bmjopen-2015-008341 [published Online First: 20151214]
13. Patel R, Chesney E, Taylor M, et al. Is paliperidone palmitate more effective than other long-acting injectable antipsychotics? *Psychol Med* 2018;48(10):1616-23. doi: 10.1017/s0033291717003051 [published Online First: 20171017]
14. Patel R, Wickersham M, Cardinal RN, et al. Natural Language Processing: Unlocking the Potential of Electronic Health Record Data to Support Transdiagnostic Psychiatric

For peer review only - http://bmjopen.bmj.com/site/about/guidelines.xhtml

Research. *Biological Psychiatry: Cognitive Neuroscience and Neuroimaging* 2022 doi: https://doi.org/10.1016/j.bpsc.2022.09.002

15. Stewart R, Soremekun M, Perera G, et al. The South London and Maudsley NHS Foundation Trust Biomedical Research Centre (SLAM BRC) case register: development and descriptive data. *BMC Psychiatry* 2009;9:51. doi: 10.1186/1471-244x-9-51 [published Online First: 20090812]
16. Fernandes AC, Cloete D, Broadbent MT, et al. Development and evaluation of a de-identification procedure for a case register sourced from mental health electronic records. *BMC Med Inform Decis Mak* 2013;13:71. doi: 10.1186/1472-6947-13-71 [published Online First: 20130711]
17. Jackson MRG, Ball M, Patel R, et al. TextHunter--A User Friendly Tool for Extracting Generic Concepts from Free Text in Clinical Research. *AMIA Annu Symp Proc* 2014;2014:729-38. [published Online First: 20141114]
18. Maudsley Biomedical Research Centre. Library of production-ready applications 2020 [Available from: https://www.maudsleybrc.nihr.ac.uk/media/325736/applications-library-v13.pdf accessed June 23, 2022.
19. Ghebreyesus TA. WHO Director-General's opening remarks at the media briefing on COVID-19 - 11 March 2020, 2020 [Available from: https://www.who.int/dg/speeches/detail/who-director-general-s- 38 opening-remarks-at-the-media-briefing-on-covid-19-11-march- 2020
20. Maudsley Biomedical Research Centre. Clinical Record Interactive Search (CRIS) 2022 [Available from: https://www.maudsleybrc.nihr.ac.uk/facilities/clinical-record-interactive-search-cris/ accessed 12 July, 2022.
21. Fusar-Poli P, Rutigliano G, Stahl D, et al. Development and Validation of a Clinically Based Risk Calculator for the Transdiagnostic Prediction of Psychosis. *JAMA Psychiatry* 2017;74(5):493-500. doi: 10.1001/jamapsychiatry.2017.0284
22. Greater London Authority. Indices of Deprivation 2020 [Available from: https://data.london.gov.uk/dataset/indices-of-deprivation) accessed June 23, 2022.
23. National Health Service. NHS Data Model and Dictionary 2022 [Available from: https://www.datadictionary.nhs.ukJune 23, 2022.
24. BNF 82 : September 2021 / Joint Formulary Committee. London: Pharmaceutical Press 2021.
25. Jackson RG, Patel R, Jayatilleke N, et al. Natural language processing to extract symptoms of severe mental illness from clinical text: the Clinical Record Interactive Search Comprehensive Data Extraction (CRIS-CODE) project. *BMJ Open* 2017;7(1):e012012. doi: 10.1136/bmjopen-2016-012012
26. Patel R, Wilson R, Jackson R, et al. Association of cannabis use with hospital admission and antipsychotic treatment failure in first episode psychosis: an observational study. *BMJ Open* 2016;6(3):e009888. doi: 10.1136/bmjopen-2015-009888

For peer review only

Associations of remote mental healthcare with clinical outcomes: a natural language processing enriched electronic health record data study protocol.

Muhammad Ahmed[1], Daisy Kornblum[2], Dominic Oliver[1], Paolo Fusar-Poli[1,2], Rashmi Patel[2,3]

[1]Department of Psychosis Studies, King's College London, Institute of Psychiatry Psychology and Neuroscience, London, UK

[2] NIHR Maudsley Biomedical Research Centre, South London and Maudsley NHS Foundation Trust, London, UK

[3]Department of Psychological Medicine, King's College London, Institute of Psychiatry Psychology and Neuroscience, London, UK

Online Supplementary Material

Supplementary Table 1 Natural language processing applications

**Application**	**Description**	**Example**	**Precision and recall**
Symptoms and signs			
Aggression	Application to identify instances of aggressive behaviour in patients, including verbal, physical and sexual aggression.	'Reported to be quite aggressive'	P=91% R=75%
Agitation	Application to identify instances of agitation.	'Very agitated at present'	P=85% R=79%
Anergia	Application to identify instances of anergia.	'Feelings of anergia'	P=95% R=89%
Anhedonia	Application to identify instances of anhedonia (inability to experience pleasure from activities usually found enjoyable).	'No evidence of anhedonia'	P=93% R=86%
Apathy	Application to extract the presence of apathy.	'Continues to demonstrate apathy'	P=93% R=86%
Arousal	Application to identify instances of arousal excluding sexual arousal.	'Hyperaroused state"	P=89% R=91%
Bad Dreams	Application to identify instances of experiencing a bad dream.	'Frequently has bad dreams	P=89% R=100%
Blunted Affect	Application to identify instances of blunted affect.	'Affect remains very blunted'	P=100% R=80%
Circumstantiality	Application to identify instances of circumstantiality.	'Loose associations and circumstantiality'	P=94% R=92%
Concrete Thinking	Application to identify instances of concrete thinking.	'No evidence of concrete thinking'	P=84% R=41%
Delusions	Application to identify instances of delusions.	'Paranoid delusions'	P=93% R=85%
Derailment	Application to identify instances of derailment.	'He derailed frequently'	P=84% R=99%

Disturbed Sleep	Application to identify instances of disturbed sleep.	'Complains of poor sleep'	P=88% R=68%
Drowsiness	Application to identify instances of drowsiness.	'She has complained of feeling drowsy'	P= 80% R=100%
Echolalia	Application to extract occurrences where echolalia is present.	'Intermittent echolalia'	P=89% R=86%
Elation	Application to identify instances of elation.	'Mildly elated in mood'	P=94% R=97%
Emotional Withdrawal	Application to identify instances of emotional withdrawal.	'Patient withdrawn'	P=85% R=96%
Flight of Ideas	Application to extract instances of flight of ideas.	'Some flight of ideas.	P=91% R=94%
Formal Thought Disorder	Application to extract occurrences where formal thought disorder is present.	'Deteriorating into a more thought disordered state'	P=83% R=83%
Grandiosity	Application to extract occurrences where grandiosity is apparent.	'Reduction in grandiosity'	P=95% R=91
Guilt	Application to identify instances of guilt.	'No longer feels guilty'	P=83% R=83%
Hallucinations (All)	Application to identify instances of hallucinations.	'Doesn't appear distressed by his hallucinations'	P=84% R=98%
Hallucinations (Auditory)	Application to identify instances of auditory hallucinations non-specific to diagnosis.	'Experiencing auditory hallucinations'	P= 80%, R=84%
Hallucinations (Olfactory/Tactile/Gustatory)	Application to extract occurrences where hallucination ((Olfactory/Tactile/Gustatory) is present.	'Seems to be having olfactory hallucinations, in relation to her tactile hallucinations'	P=78%, R=68%
Hallucinations (Visual)	Application to extract occurrences where visual hallucination is present. Visual hallucinations may be due to a diagnosis of psychosis/schizophrenia or may be due to other causes, e.g., due to substance abuse.	'Experiencing visual hallucination'	P=91%, R=96%
Helplessness	Application to identify instances of helplessness.	'Ideas of helplessness'	P=93% R=86%
Hopelessness	Application to identify instances of hopelessness.	'Says feels hopeless'	P=90% R=95%
Hostility	Application to identify instances of hostility.	'Increased hostility and paranoia'	P=89%, R=94%
Insomnia	Application to identify instances of insomnia.	'Complaining of insomnia'	P=89%, R=94%
Irritability	Application to identify instances of irritability.	'Became irritable'	P=100% R=83%
Loss of Coherence	Application to identify instances of incoherence or	'Patient was incoherent'	P=98% R=95%

	loss of coherence in speech or thinking.		
Low Energy	Application to identify instances of low energy.	'Decreased energy'	P=72% R=67%
Mood Instability	This application identifies instances of mood instability.	'Expressed fluctuating mood'	P=100% R=70%
Mutism	Application to identify instances of mutism.	'Did not respond any further and remained mute'	P=91% R=75%
Negative Symptoms	Application to identify instances of negative symptoms.	'Diagnosis of schizophrenia with prominent negative symptoms'	P=86% P=95%
Nightmares	Application to identify instances of nightmares.	'Unsettled sleep with vivid nightmares'	P=89% R=100%
Paranoia	Application to identify instances of paranoia. Paranoia may be due to a diagnosis of paranoid schizophrenia or may be due to other causes, e.g., substance abuse.	'Vague paranoid ideation'	P=86%, R=94%
Passivity	Application to identify instances of passivity.	'Patient describes experiencing passivity'	P=89% P=100%
Persecutory Ideation	Application to identify instances of ideas of persecution.	'Marked persecutory delusions'	P=80% R=96%
Poor Appetite	Application to identify instances of poor appetite (negative annotations).	'Normal appetite'	P=83% R=71%
Poor Concentration	Application to identify instances of poor concentration.	'Finds it hard to concentrate'	P=84% R=60%
Poor Insight	Applications to identify instances of poor insight.	'Limited insight'	P=87% R=70%
Poor Motivation	This application aims to identify instances of poor motivation.	'Struggling with motivation'	P=95% R=38%
Poverty of Speech	Application to identify poverty of speech.	'Continues to display negative symptoms including poverty of speech'	P=87% R=85%
Poverty of Thought	Application to identify instances of poverty of thought.	'Evidence of poverty of thought'	P=95%, R=93%
Social Withdrawal	Application to identify instances of social withdrawal.	'Very isolated and socially withdrawn'	P=60% R=86%
Stupor	Application to identify instances of stupor. This includes depressive stupor, psychotic stupor, catatonic stupor, dissociative stupor and manic stupor.	'Man with stuporous catatonia'	P=88% R=87%
Suicidal Ideation	Application to identify instances of suicidal ideation	'No suicidal ideation disclosed'	P=81% R=87%

For peer review only

	- thinking about, considering, or planning suicide.		
Tangentiality	Application to identify instances of tangentiality.	'Evidence of tangential speech'	P=99% R=90%
Tearfulness	Application to identify instances of tearfulness.	'Appeared tearful'	P=100% R=94%
Thought Block	Application to identify instances of thought block.	'Showed some thought block'	P=91% R=75%
Thought Broadcast	Application to identify instances of thought broadcasting.	'Patient describes experiencing thought broadcasting'	P=86% R=92%
Thought Insertion	Application to identify instances of thought insertion.	'No thought insertion'	P=81% R=96%
Thought Withdrawal	Application to identify instances of thought withdrawal.	'Presence of thought withdrawal'	P=90% R=88%
Waxy Flexibility	Application to identify instances of waxy flexibility. Waxy flexibility is a psychomotor symptom of catatonia as associated with schizophrenia, bipolar disorder, or other mental disorders which leads to a decreased response to stimuli and a tendency to remain in an immobile posture.	'Presents as catatonic with waxy flexibility'	P=80% R=86%
Weight Loss	Application to identify instances of weight loss.	'Significant weight loss'	P=90% R=88%
Worthlessness	Application to identify instances of worthlessness.	'Feeling worthless'	P=88% P=86%
Contextual factors			
Asthma	Application to identify patients with diagnosis of asthma.	'Suffered from an asthma attack'	P=95% R=84%
Bronchitis	Application to identify patients with diagnosis of bronchitis.	'Past diagnosis: chronic obstructive airway disease'	P=85% R=48%
Cough	Application to identify instances of coughing.	'Denied any coughing or shortness of breath'	P=83% R=80%
Crohn's Disease	Application to identify patients with diagnosis of Crohn's disease.	'Has Crohn's disease'	P=94% R=78%
Hypertension	Application to identify patients with diagnosis of hypertension or high blood pressure.	'History of hypertension'	P=94% R=94%
Rheumatoid Arthritis	Application to identify patients with diagnoses of rheumatoid arthritis.	'Pain due to her rheumatoid arthritis'	R=91% R=86%
Amphetamine	To identify instances of amphetamine use.	'Has not used amphetamine for the last week'	P=80% R=84%
Cannabis	To identify instances of cannabis use.	'No cannabis use'	P=77% R=93%

Cocaine/Crack Cocaine	To identify instances of cocaine or crack cocaine use.	'He has stopped taking cocaine'	P=84% R=97%
MDMA	Application to identify instances of MDMA use.	'First took MDMA at the age of 15'	P=100% R=99%
Tobacco Smoking	This application distinguishes between people who are a) current smokers, b) current non-smokers (ever smoked) and c) non-smokers.	'Doesn't smoke'	P=81% R=74%
Lives Alone	Application to identify instances of living alone.	'Lives on her own'	P=77% R=83%
Domestic Violence;	Application to identify instances of domestic violence.	'Victim of DV'	P=86% R=93%
Loneliness	Application to identify instances of loneliness.	'Patient is lonely'	P= 87% R= 100%
Interventions			
Cognitive Behavioural Therapy (CBT)	An application to identify instances of delivered sessions of Cognitive Behavioural Therapy (CBT).	'Session of CBT'	P=99% R=82%
Family Intervention	The application identifies instances of family intervention delivery.	"Family therapy"	P=77% R=87%
Medication	The application identifies instances of medication prescription.	'Quetiapine'	Antipsychotics: P=97%-99%, R=39%
Outcomes and Clinical Status			
Blood Pressure (BP)	Application to identify instances of blood pressure scores in the format of overall score, systolic blood pressure score and diastolic blood pressure score.	'140/90mmHg'	P=98% R=96%
Body Mass Index (BMI)	Application to identify body mass index (BMI) scores.	'Body Mass Index is 22.9'	P=89% R=78%
HbA1c	Application to identify HbA1c results.	'HbA1c was 40mmol/mol'	P=89% R=93%
Mini-Mental State Examination (MMSE)	This app identifies MMSE scores.	'25/30'	P=93% R=94%
Diagnosis	Application to extract instances of diagnosis.	'Schizophrenia'	F20: P=100%, R=65%
Treatment-Resistant Depression	Application to identify instances of treatment-resistant depression.	'History of treatment resistant depression'	P=77% R=95%
Bradykinesia (Dementia)	To identify instances of bradykinesia in the context of dementia.	'Moderate bradykinesia'	P=91% R=84%
Tremor (Dementia)	Application to identify instances of tremor in patients with dementia.	'With a degree of resting tremor'	P=83%, R=92%

For peer review only
